# BK channels sustain neuronal Ca^2+^ oscillations to support hippocampal long-term potentiation and memory formation

**DOI:** 10.1007/s00018-023-05016-y

**Published:** 2023-11-21

**Authors:** Thomas Pham, Tamara Hussein, Dila Calis, Helmut Bischof, David Skrabak, Melanie Cruz Santos, Selina Maier, David Spähn, Daniel Kalina, Stefanie Simonsig, Rebekka Ehinger, Bernhard Groschup, Marlies Knipper, Nikolaus Plesnila, Peter Ruth, Robert Lukowski, Lucas Matt

**Affiliations:** 1 Department of Pharmacology, Toxicology and Clinical Pharmacy, Institute of Pharmacy, University of Tübingen, Tübingen, Germany; 2 Department of Otolaryngology, Head and Neck Surgery, Molecular Physiology of Hearing, Tübingen Hearing Research Centre, University of Tübingen, Tübingen, Germany; 3 Laboratory of Experimental Stroke Research, Institute for Stroke and Dementia Research (ISD), University Hospital, Ludwig-Maximilians-University Munich (LMU), Munich, Germany; 4 Munich Cluster for Systems Neurology (SyNergy), Munich, Germany

**Keywords:** Large-conductance Ca^2+^- and voltage-activated potassium channel, BK, Synaptic plasticity, Long-term potentiation (LTP), Intracellular K^+^ dynamics

## Abstract

Mutations of large conductance Ca^2+^- and voltage-activated K^+^ channels (BK) are associated with cognitive impairment. Here we report that CA1 pyramidal neuron-specific conditional BK knock-out (cKO) mice display normal locomotor and anxiety behavior. They do, however, exhibit impaired memory acquisition and retrieval in the Morris Water Maze (MWM) when compared to littermate controls (CTRL). In line with cognitive impairment in vivo, electrical and chemical long-term potentiation (LTP) in cKO brain slices were impaired in vitro. We further used a genetically encoded fluorescent K^+^ biosensor and a Ca^2+^-sensitive probe to observe cultured hippocampal neurons during chemical LTP (cLTP) induction. cLTP massively reduced intracellular K^+^ concentration ([K^+^]_i_) while elevating L-Type Ca^2+^ channel- and NMDA receptor-dependent Ca^2+^ oscillation frequencies. Both, [K^+^]_i_ decrease and Ca^2+^ oscillation frequency increase were absent after pharmacological BK inhibition or in cells lacking BK. Our data suggest that L-Type- and NMDAR-dependent BK-mediated K^+^ outflow significantly contributes to hippocampal LTP, as well as learning and memory.

**Supplementary Information:**

The online version contains supplementary material available at 10.1007/s00018-023-05016-y.

## Introduction

Mutations in *KCNMA1*, the human gene encoding the pore forming α subunit of the large-conductance calcium ion (Ca^2+^)- and voltage-activated potassium ion (K^+^) channel BK (maxi K^+^, K_Ca_1.1, Slo1) are linked to a growing number of clinically relevant phenotypes. Besides movement disorders and epileptic seizures, these mutations are accompanied by cognitive impairments like developmental delay and intellectual disability [1], which are also linked to autism spectrum disorders and schizophrenia [2, 3].

Throughout the mammalian central nervous system [4], the ubiquitously expressed BK was found in pre- as well as postsynaptic locations [5, 6], where it associates closely with voltage-gated Ca^2+^ channels [7] and *N*-methyl-d-aspartate (NMDA)-type glutamate receptors (NMDAR; [8]). These act as Ca^2+^ sources to provide high local [Ca^2+^]_i_ of more than 10 µM necessary for BK activation in the physiologically relevant voltage range of − 50 to 0 mV [7, 9, 10], reviewed in [11]. In presynaptic terminals, massive K^+^ outflow in response to BK activation and the ensuing membrane hyperpolarization limits synaptic transmission by reducing Ca^2+^ influx through voltage-gated Ca^2+^ channels [12]. In some postsynaptic cells, BK was described to decrease activity levels through hyperpolarization-mediated inhibition of voltage-gated cation channels [13]. In other cell populations like hippocampal CA1 pyramidal cells, however, BK increases firing rate by contributing to the fast repolarization after individual action potentials (AP) [14–16].

Brain development [17] as well as learning and memory aspects of cognition essentially depend on NMDAR-dependent plasticity of α-amino-3-hydroxy-5-methyl-4-isoxazolepropionic acid (AMPA)-type glutamate receptor (AMPAR)-dependent glutamatergic transmission [18]. High frequency stimulation (HFS) of AMPAR provokes postsynaptic Ca^2+^ influx through NMDAR and voltage-activated L-Type Ca^2+^ channels (LTCC; [19, 20]). The resulting increase in [Ca^2+^]_i_ strengthens synapses by increasing AMPAR conductance and exocytosis-mediated integration of additional postsynaptic AMPAR. This process of synaptic strengthening is called long-term potentiation (LTP). The converse mechanism termed long-term depression (LTD) reduces synaptic strength by AMPAR endocytosis [21].

Synaptic plasticity in the form of LTP and LTD has been most intensively studied in the hippocampus, a brain region involved in spatial and declarative learning and memory [22], where it is compromised in different models of cognitive impairment [23–26]. So far, however, little is known about the role of postsynaptic BK in hippocampal synaptic plasticity. Reportedly, BK-mediated afterhyperpolarization (fAHP) in hippocampal CA1 pyramidal cells is modulated in response to learning [27]. Additionally, global BK knock-out mice (BK KO or BK^−/−^) suffer from delayed acquisition of hippocampus-dependent spatial memory in the Morris Water Maze (MWM) [28].

Combining behavioral, electrophysiological and biochemical approaches, we found impaired hippocampal learning and LTP in hippocampus-specific conditional BK KO (cKO) mice as well as reduced Ca^2+^ oscillations and K^+^ loss in dissociated neurons lacking BK function during chemically induced LTP (cLTP) in vitro. Hence, we propose that BK-mediated hyperpolarizing K^+^-currents support postsynaptic Ca^2+^ activity necessary for hippocampal LTP expression.

## Materials and methods

### Animals

All experimental studies were approved by the local ethics Committee for Animal Research (Regierungspräsidium Tübingen, Germany) and performed according to the guidelines of the German Animal Welfare Act. Animals were kept in standardized cages on a 12/12 h light/dark cycle (lights on 6 a.m.–6 p.m.) under controlled temperature and humidity with access to food and water ad libitum.

8–12 weeks-old animals of both sexes used for characterization by PCR, Western blot and immunofluorescence as well as behavioral, electrophysiological and cLTP experiments were generated by mating the T29-1 transgenic subline expressing Cre recombinase under control of the CaMKII promoter in CA1 pyramidal neurons (B6.Cg-Tg(Camk2a-cre)T29-1Stl/J, JAX #005359) [29] with mice heterozygous for the floxed (BK^fl/+^; B6-Kcnma1^tm2.1Ruth^) or the BK KO allele (BK^−/+^; (B6-Kcnma1^*tm1Ruth*^) [30]. Subsequent intercrossing to floxed BK (BK^fl/fl^) mice produced age- and litter-matched CA1 pyramidal neuron-specific conditional BK knockouts (cKO, *genotype*: Cre^tg/+^; BK^fl/−^) and respective controls (CTRL, *genotype*: Cre^tg/+^; BK^fl/+^). Primary hippocampal neurons for cultures were obtained from homozygous P0–P1 wildtype (BK^+/+^) or KO (BK^−/−^) animals bred from either homozygous BK^+/+^ or heterozygous (BK^−/+^) breeders, respectively. Genetic status of neuronal cultures prepared from individual pups was determined by genotyping PCR of biopsies obtained from originating animals. Primer sequences are listed in Table S6.

### Antibodies and reagents

All primary antibodies used in the present study are listed in Table S7. All secondary antibodies were isotype-specific and conjugated to Alexa Fluor 488- or Alexa Fluor 555 (Thermo Fisher Scientific). *dl*-2-Amino-5-phosphonopentanoic acid sodium salt (AP-5, #105), Forskolin (#1099), and Paxilline (#2006) were from Tocris. Nifedipine (#N7634), Picrotoxin (#P1675) and Rolipram (#R6520) were from Sigma-Aldrich. DNA oligonucleotide primers used in this study are listed in Table S6. If not specifically stated, all other reagents were of standard quality and from the usual vendors.

### DNA Extraction and PCR

DNA was isolated from freshly dissected hippocampi of T29.1-Cre^tg^; BK^fl/+^ using High Pure PCR Template Preparation Kit (Roche #11796828,001) according to manufacturer specifications. BKα-null allele (132 bp) was amplified using KAPA HotStart Mouse-Genotyping Kit (Roche #KK7352) and BK forward-1 5′-TGG TCT TCT TCA TCC TCG GG-3′; BK forward-2 5′-AAG GGC CAT TTT GAA GAC GTC-3′ and BK reverse 5′-CCA GCC ACG TGT TTG TTG G-3′ primers to confirm hippocampus specific recombination of BK.

### Immunofluorescence

Mice were euthanized with CO_2_ and transcardially perfused with 20 mL Dulbecco's Balanced Salt Solution (DPBS, Thermo Scientific #14190144) followed by 20 mL 4% (wt/vol) paraformaldehyde in DPBS to fix the tissue. Brains were isolated, snap frozen in -40 to -60 °C cold isopentane and stored at − 80 °C. Brains were transferred to − 20 °C 2 h before 8 µm thick coronal sections were obtained. Slices from CTRL, cKO and BK^−/−^ were collected on the same glass slides (epredia Superfrost™ Plus Adhesion Microscope Slides #J1800AMNZ) together with additional sections from BK^+/+^ that served as positive controls. Blocking buffer (BB) consisting of DPBS supplemented with 2% Glycerol, 5% NGS, 0.3% Triton-X-100, 2% (wt/vol) BSA and 50 mM NH_4_Cl was used for permeabilization and blocking of unspecific antibody binding. Primary antibodies against BK were diluted 1:1000 in BB and incubated overnight at 4 °C. Sections were then washed thrice with 0.01% Triton-X-100 in DPBS and blocked again with BB for 1 h before incubation with secondary antibody (1:2500) and Hoechst 33342 (1:1000) for 2 h. After three washes with 0.01% Triton-X-100 in DPBS, DPBS and water, sections were mounted with Permafluor (Fisher Scientific, #TA-030-FM). Primary antibodies are listed in Table S7.

### Tissue lysis and western blot

Upon dissection, tissues were suspended in RIPA buffer (in mM: 50 Tris, 150 NaCl, 5 EGTA, 10 EDTA, final pH 7.4) containing protease inhibitors (in μg/ml; 1 phenylmethanesulfonyl fluoride, 1 pepstatin A, 10 leupeptin, 20 aprotinin), 1% NP-40, 10% glycerol, 0.05% sodium dodecyl sulfate (SDS), 0.4% deoxycholate and phosphatase inhibitors (Sigma Phosphatase Inhibitor Cocktail 2 and 3) and homogenized with a hand disperser (Polytron).

Protein concentration was measured using BCA assay (Thermo Scientific #23227). 80 µg total protein were incubated with SDS sample buffer at 95 °C for 5 min, separated by electrophoresis in 10% polyacrylamide gels, transferred to polyvinylidene fluoride (PVDF) membranes (Merck #IPFL00010) using a semi-dry blotting system (Carl Roth, 110 mA per membrane for 90 min), probed with the indicated primary antibodies (Table S7) and detected with fluorescently labelled secondary antibodies. Immunosignals were visualized using an Amersham Imager 600 (General Electrics). To simultaneously label multiple proteins of different molecular weight on the membrane using antibodies, the membranes were cut with a little safety margin, according to the size of the proteins of interest. The contrast of the displayed blots was increased to improve visibility of the bands. Importantly, however, densiometric quantification using ImageJ was performed with unprocessed raw images.

### Blinding procedures

During the in vivo experiments (beam-walk, open-field and water-maze) as well as electrophysiology, the experimenter was unaware of the animals’ genotype. To mask the genotypes, a person other than the experimenter blinded genotypes prior to each experiment by assigning a randomized number to each mouse that did not provide the experimenter with any information about the genotype. Only after data analysis, the experimenter was informed about genotypes.

### Beam-Walk

As previously described [31], mice were trained for 3 consecutive days with four trials per day on a rectangular beam of 1 m length with 12 mm edge length suspended in 0.5 m height. The area below the beam was cushioned to prevent fall injuries. On test day, latency to traverse rectangular (28, 12 and 5 mm edge length) and circular beams (28, 17 and 11 mm diameter) was recorded and analyzed offline by an observer unaware of the genotype for latency to cross beam, hind paw foot slips and number of falls.

### Open-Field

Open-Field was performed as previously described [31]. Mice were recorded while freely exploring a circular arena with a diameter of 112 cm for 30 min using a suspended camera. Latency to enter border zone (15 cm from the edge), relative time spent in the border zone, total  distance travelled, resting time and mean speed were quantified and analyzed using Smart 3.0 tracking software (Panlab). Additionally, the number of rearings during the first 5 min was quantified.

### Spatial acquisition and reversal (Water-Maze)

As previously described [31], a 112 cm diameter pool was filled with 30 cm of water (25 °C) made opaque by milk powder. A transparent cylindrical escape platform of 12 cm diameter was submerged 0.5 cm beneath the water surface. The maze was virtually divided into 4 quadrants NE, SE, SW and NW. Swimming trials were recorded with a camera suspended above center of the pool and processed via Smart 3.0 tracking software (Panlab). Mice were trained for 5 consecutive days with four trials per day to find the platform submerged in the NW quadrant using visual cues. For every trial, mice were released into the pool from a different starting location. On day 6, the platform was removed for probe trial. Mice were released into SE quadrant opposite of the target and their movement was tracked for 60 s. After acquisition probe trial, a reversal phase was performed in which the cognitive flexibility was queried. For this purpose, the platform was positioned in the opposite SE quadrant and insertion locations were mirrored. Training days and number of trials as well as probe trial were otherwise performed identically to the initial acquisition phase.

### Electrophysiology

Extracellular fEPSP were recorded as previously described [38]. In brief, brains were quickly isolated and immersed in ice-cold slicing buffer (in mM: 127 NaCl, 1.9 KCl, 26 NaHCO_3_, 1.2 KH_2_PO_4_, 10 D-glucose, 2 MgSO_4_, and 1.1 CaCl_2_, saturated with 5% CO_2_ and 95% O_2_, final pH 7.4). 400 µM thick forebrain sections were coronally cut on a vibrating microtome (Leica VT 1000S) and stored in artificial cerebrospinal fluid (ACSF, in mM: 127 NaCl, 1.9 KCl, 26 NaHCO_3_, 1.2 KH_2_PO_4_, 2.2 CaCl_2_, 1 MgSO_4_ and 10 D-glucose, oxygenated with 95% O_2_ plus 5% CO_2_, final pH 7.4) at 30 °C for at least 1 h before remaining at room temperature until measurement. Sections were transferred to a measurement chamber constantly perfused with oxygenated warm ACSF at 30 °C. Stimulation (bipolar concentric, TM53CCINS, WPI) and recording (ACSF-filled glass pipettes, 2–3 MΩ) electrodes were positioned in the *stratum radiatum* to measure Schaffer collateral fEPSPs. Signals were amplified using an Axopatch 200B amplifier (Molecular Devices), digitized by a LIH 8 + 8 (HEKA) at 5 kHz and recorded using WinWCP from the *Strathclyde Electrophysiology Suite*. Stimuli (100 µs) were applied through a stimulus isolator (WPI). For each individual slice the strength of the stimulation (typically between 50 and 100 μA) was chosen to evoke approximately 50% of the maximal response, defined by initial fEPSP slope. LTP was induced by high-frequency stimulation (HFS; 100 Hz, 1 s) at the same intensity as baseline. Baseline and LTP levels were determined by average fEPSP initial slopes from the period between −10 to 1 min before and 45 and 60 min after HFS, respectively. Paired Student's T-test was used to determine whether LTP was induced within a genotype (mean baseline vs. mean LTP levels). Two-way ANOVA was used to compare LTP levels (45 to 60 min after HFS) between CTRL and cKO. Before each LTP measurement, input–output ratio (IOR) was determined for stimulus intensities between 25–150 µA and paired-pulse facilitation (PPF) for inter-stimulus intervals of 10, 20, 50, 100, 200, and 500 ms (with the same intensity as for the LTP measurement) for each slice. The stimulation interval was 1/15 s, with 4 traces being averaged to one data point. Data were analyzed and processed using Clampfit 10 (Molecular Devices) and Microsoft Excel. Statistics and visualization were performed with GraphPad Prism. Two-way ANOVA with Sidak's multiple comparison was used to compare IOR and PPF between CTRL and cKO.

### Chemically induced LTP (cLTP)

Freshly isolated brains were immediately transferred into ice cold sucrose dissection buffer (SACSF, in mM: 254 sucrose, 1.9 KCl, 1.2 KH_2_PO_4_, 26 NaHCO_3_, 10 D-glucose, 2 MgSO_4_ and 1.1 CaCl_2_, saturated with 5% CO_2_ and 95% O_2,_ final pH 7.4). Hippocampi were isolated and cut into 350 µM transversal sections using a McIlwain Tissue chopper and incubated in oxygenated ACSF (see above) for 30 min at room temperature. cLTP was induced by adding 20 µM forskolin, 0.1 µM rolipram 50 µM picrotoxin for 10 min in the presence or absence of 5 µM paxilline [32]. This treatment increases cAMP levels [32] and subsequently increases network activity leading to tetanic-like bulk stimulation potentiating a majority of excitatory synapses [33]. Stimulation was terminated by subsequently storing slices in oxygenated ACSF for an additional 10 min.

cLTP induction was evaluated by Western immunoblot using the indicated antibodies (Table S7) as described above. After detection of phospho-specific GluA1 signals, membranes were stripped using Re-blot Plus Strong Antibody Stripping Solution (Millipore) before incubation with GluA1-specific antibodies and detection with secondary antibodies labelled with a fluorescent dye different from the one used for detection of phospho signals (all primary antibodies are listed in Table S7).

### Primary dissociated hippocampal neurons

Primary hippocampal neurons were cultured from P0 BK^+/+^ and BK^−/−^ as previously described [34]. In summary, hippocampi were isolated in dissection medium (DM) consisting of Hank’s Balanced Salt Solution (Invitrogen #14175095) supplemented with 1 mM sodium pyruvate, 0.1% (wt/vol) Glucose and 10 mM HEPES) and cleared of meninges. After washing thrice with DM, hippocampi were trypsinized (0.25% (wt/vol) in DM for 20 min at 37 °C) and subsequently incubated with 0.1% (wt/vol) Deoxyribonuclease I (Sigma #DN25) for 5 min at room temperature. After washing twice with DM, trypsin was deactivated by washing twice with plating medium (PM) consisting of BME medium (Invitrogen #21010046) supplemented with 10% FCS, 0.45% (wt/vol) glucose, 1 mM sodium pyruvate, 2 mM glutamine and 100 U/ml penicillin/streptomycin. Neurons were dissociated by triturating several times using a fire-polished glass pipette, counted and seeded (700,000 cells) onto 30 mm circular poly-l-lysine coated (Sigma #P2636) glass coverslips. After 2 h, PM was replaced with maintenance medium (MM; Neurobasal; Invitrogen #21103049) supplemented with B-27 (Invitrogen #17504044), 2 mM glutamine and 100 U/ml penicillin/streptomycin. Cells were maintained at 37 °C in a humidified environment with 5% CO_2_/95% air. Half of the MM was replaced every 3–4 days.

### K^+^ imaging

FRET-based recordings of [K^+^]_i_ were performed as previously described [35]. Neurons were transduced after 7 days in vitro (DIV) at a multiplicity of infection (MOI) of 100 with an adeno associated virus -DJ/8 vector system encoding a cytosol targeted K^+^ sensitive lc-LysM GEPII 1.0 FRET-based biosensor [36] under control of a CAG promoter. At 9 DIV, coverslips were mounted in a PC30 perfusion chamber (NGFI GmbH, Graz, Austria), perfused with imaging buffer (IB, in mM: 126.5 NaCl, 5 KCl, 2 CaCl_2_, 2 MgCl_2_, 10 HEPES, 30 D-glucose, 10 sodium pyruvate, final pH 7.4) through a gravity-based perfusion system (NGFI GmbH, Graz, Austria) which was also used for drug application. Single cell live imaging was conducted on a Zeiss Observer Z.1 (Wetzlar, Germany) with an external light source (2200114 LED-Hub, Omicron Laserage, Rodgau-Dudendorf, Germany) and a Plan-Neofluar 40x/1.30 Oil immersion objective as previously described [37]. Emissions were simultaneously collected at 480 and 535 nm using an Optosplit II (Cairn Research, Faversham, UK). The LED hub (Omicron Laserage, Rodgau-Dudendorf, Germany) featured a 340 nm, 380 nm and 455 nm, LED with 340×, 380× and 427/10 bandpass filters, respectively (AHF Analysentechnik, Tübingen, Germany). Emissions were recorded using a 459/526/596 dichroic with a 475/543/702 emission filter (AHF Analysentechnik, Tübingen, Germany). Images were recorded using a pco.panda 4.2 bi sCMOS camera (PCO, Kelheim, Germany). VisiView software (Visitron Systems, Puchheim, Germany) was used to acquire images and to control the microscope. During live cell imaging, drugs were applied 10 min prior to the start of the recording. Background subtraction was performed using a background ROI. The ratio, which is proportional to [K^+^]_i_ levels, was calculated from the corrected values of the YFP images to those of CFP. First 5 min of basal FRET ratio signal were used for normalization.

### Ca^2+^ imaging

Ca^2+^-sensitive live cell imaging was conducted as described before [38]. 9 DIV neurons were loaded with 2.5 µM of the ratiometric dye Fura-2AM (AAT Bioquest, Sunnyvale, USA) in IB for 20 min at 37 °C followed by a washing step in IB for 10 min at room temperature. Excitation at 340 nm (Ca^2+^ bound to Fura-2) and 380 nm (Ca^2+^ free) induced emissions which were recorded on the same setup as K^+^ signals. Background subtraction was performed using a background ROI. The ratio, which is proportional to [Ca^2+^]_i_ levels, was calculated from the corrected values of the images at 340 nm to those taken at 380 nm. Only spikes with at least 10% of the height of the glutamate peak were considered in the analysis.

### Membrane potential imaging

Membrane potential was visualized by live cell fluorescence imaging using membrane potential sensitive dye Bis-(1,3-dibutylbarbituric acid)trimethine oxonol (DiBAC_4_(3)) [39]. 9 DIV neurons were loaded with a 1:40,000 dilution of a 10 mg/mL stock of DiBAC_4_(3) for 30 min at RT in IB. During recordings DiBAC_4_(3) in the indicated concentration was present in all buffers used. cLTP induction, recording of induced emissions at 516 nm and background subtraction by background ROI were performed as described for K^+^- and Ca^2+^ -sensitive recordings.

### Statistical analysis

**Fig. 1:**
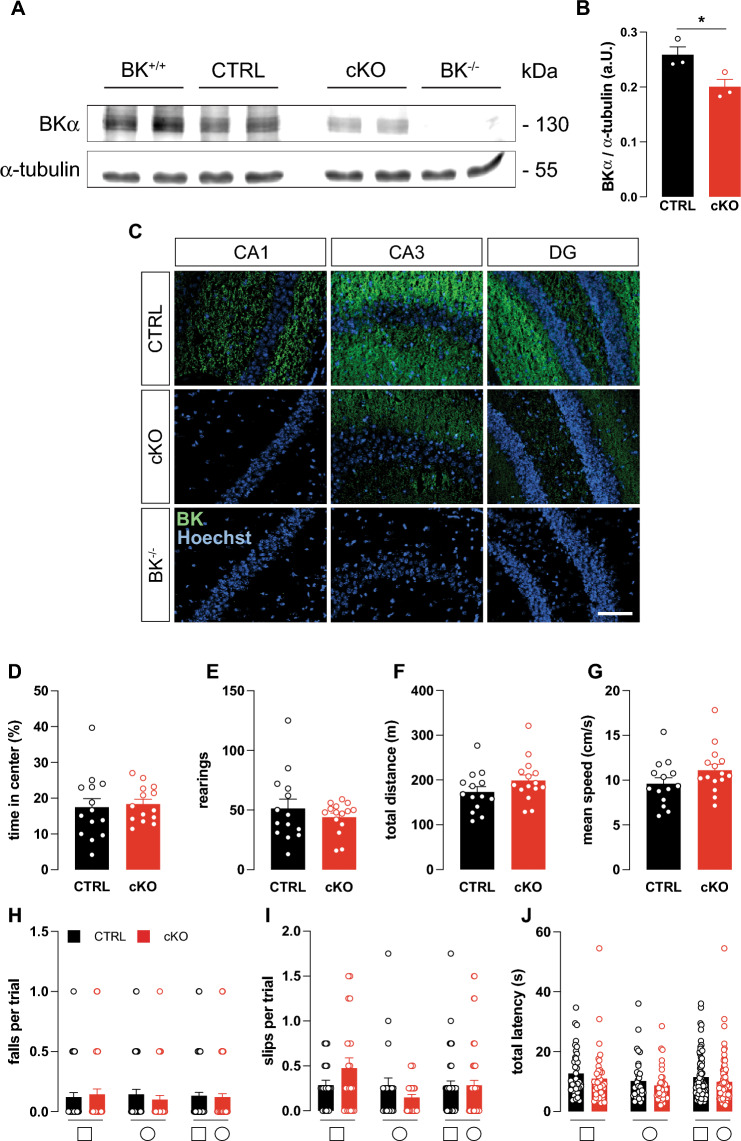
Characterization of conditional hippocampus-specific BK KO mice. **A** Representative immunoblot of whole hippocampal lysates from wildtype (BK^+/+^), conditional BK control (CTRL), conditional (cKO) and global BK KO (BK^−/−^) mice probed using BKα-specific antibodies revealed that BK protein levels were comparable between BK^+/+^ and CTRL, but significantly reduced in cKO. Compared to CTRL no BKα immunoreactivity was obtained in BK^−/−^ samples. α-Tubulin immunoreactivity serves as loading control. **B** Quantification of BK protein band intensities normalized to α-tubulin. n = 3 independent samples from N = 3 mice. **C** Representative immunofluorescence labelling of cryosections from CTRL, cKO and BK^−/−^ mice using specific antibodies against the pore forming α-subunit of BK (BKα, green). Nuclei were stained with Hoechst 33342 (blue). In CTRL, BKα immunosignals were detected in all hippocampal regions. In cKO, BK immunosignals were virtually absent in CA1, but were present in CA3 and DG. No BK immunosignals were detected in BK^−/−^ mice. n = 3 protein samples obtained from N = 3 mice per genotype. Scale bar is 100 µm. **D**–**G** During 30 min of observation in the open field experiment, time spent in center of the arena (**D**), number of rearings (**E**), total distance travelled (**F**) and mean velocity (**G**) did not differ between cKO and CTRL (N = 14). **H**–**J** cKO and CTRL performed similar in the beam walk test as assessed by falls per trial (**H**), missteps per trial (**I**) and latency to cross the bar (**J**). Diagrams depict averages of all bar diameters of the square (□, left), round (○, middle) or both (□○, right) cross-sections indicated below (N = 10–14). Statistics: Unpaired Student's t-test (**D–G**), Two-way ANOVA with Sidak's multiple comparison test (**H**–**J**). All bar diagrams presented as means ± SEM. See also Fig. S1 and Table S1

**Fig. 2:**
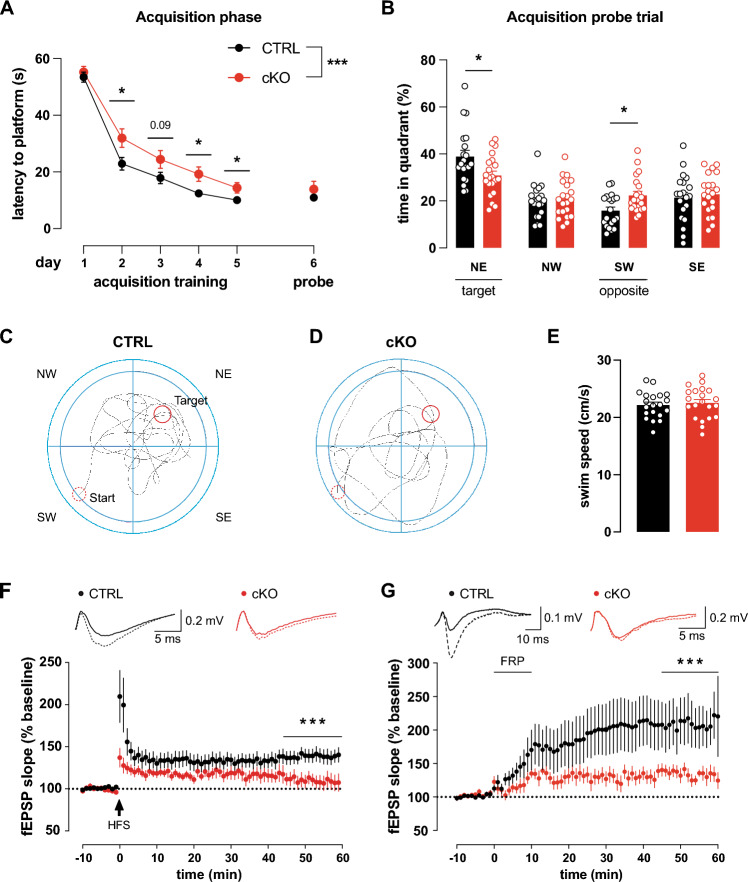
Impaired MWM performance and hippocampal LTP in cKO. **A**–**C** Compared to CTRL (N = 20), cKO (N = 21) exhibit delayed learning and reduced memory in the MWM task. **A** Mean latencies to reach the hidden platform or original platform position over 5 training days (4 training sessions per day). Latency was similar on day 1 but decreased quicker in CTRL than cKO during the following trainings days. Since the hidden platform was removed, latency during probe trial on day 6 was measured for reaching the former platform position. The latency of the probe is offset, as it is not part of the acquisition phase. **B** CTRL but not cKO showed significant target quadrant (NE) preference during probe trial and also significantly avoided the opposing quadrant (SW). **C** Representative paths traveled by the CTRL and **D** cKO during acquisition probe trial. Start and finish indicated by dashed and solid red circle, respectively. **E** Swim speed of CTRL (N = 20) and cKO (N = 21) during acquisition probe trial were comparable. **F** Schaffer-collateral fEPSP initial slopes recorded from forebrain slices. 100 Hz 1 s high frequency stimulation (HFS) induced significantly more LTP in CTRL (n = 7 slices from N = 4 animals) than cKO (n = 8 slices from N = 4 animals). Top: Representative traces before (solid) and after (dashed) LTP induction. **G** Schaffer-collateral fEPSP initial slopes recorded from forebrain slices. 10 min perfusion of 20 µM forskolin, 50 µM picrotoxin and 0.1 µM rolipram (FRP) induced significantly more cLTP in CTRL (n = 10 slices from N = 4 animals) than cKO (n = 10 slices from N = 5 animals). Top: Representative traces before (solid) and after (dashed) LTP induction. Statistics: Two-way ANOVA (**F**, **G**) with Sidak's multiple comparison test (**A**,** B**), unpaired Student's t-test (**E**). All bar diagrams presented as means ± SEM. See also Fig. S2 and Table S2

**Fig. 3:**
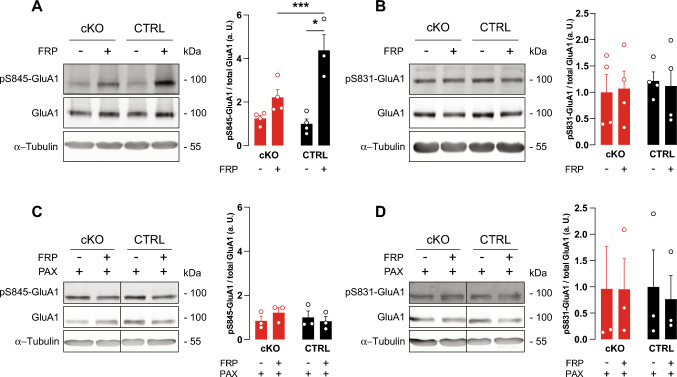
BK deficiency precludes GluA1 S845 phosphorylation after LTP induction. Acute hippocampal slices from 8–12 weeks-old mice were treated with vehicle or 20 µM forskolin, 0.1 µM rolipram and 50 µM picrotoxin for 10 min to chemically elicit LTP in the presence or absence of the BK channel blocker PAX (5 µM). 10 min after cLTP induction, slices were lysed for immunodetection of phosphorylation at GluA1 residues S845 and S831. Blots were stripped and reprobed for total GluA1. Loading control: α-Tubulin. Representative blots on the left, densitometric quantification on the right. Relative expression was normalized to the mean of the respective untreated CTRL group not stimulated by cLTP to better represent the ratios. **A** cLTP significantly increased GluA1 S845 phosphorylation in CTRL but not cKO (N = 4 animals per genotype), **B** while S831 phosphorylation levels were not altered. **C** Presence of PAX precludes changes in S845 phosphorylation levels. **D** S831 phosphorylation remains unchanged after cLTP in both CTRL (N = 3) and cKO (N = 3) also in the presence of PAX. Statistics: Two-way ANOVA with Sidak's multiple comparison test. All bar diagrams presented as means ± SEM. See also Table S3

**Fig. 4:**
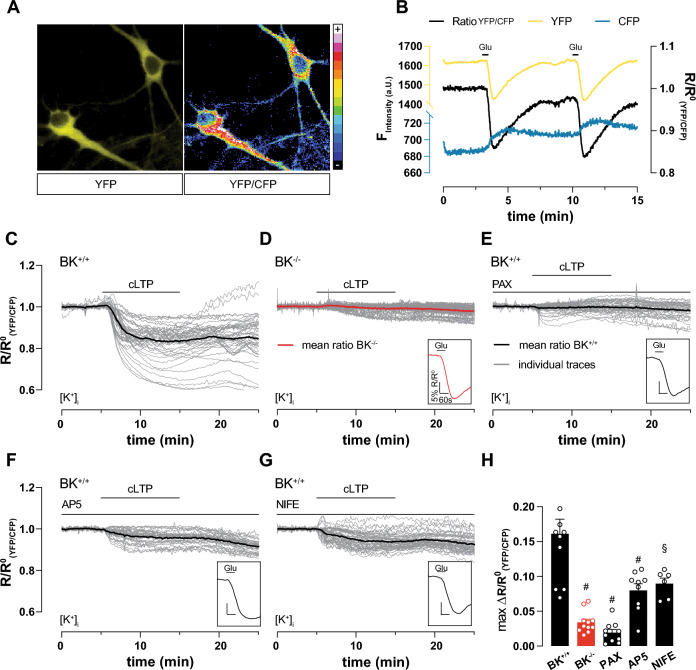
cLTP induction causes massive K^+^ outflow depending on BK, NMDAR and LTCC. Live imaging of 9 DIV hippocampal neuronal cultures (representative cells in **A**), virally transduced at 7 DIV with the FRET-based K^+^-sensitive sensor (GEPII), allowed single-cell live recording of [K^+^]_i_ in response to external stimuli. (**B**) Representative time course of ratio between single fluorescence intensities (YFP/CFP, black) highlights reduction of [K^+^]_i_ after repeated application of 20 µM glutamate. Single fluorescence intensities shown in yellow and blue as indicated. **C**–**G** Individual (gray) and averaged (black or red in (**D**)) YFP/CFP in response to cLTP induction in neurons. Glutamate (20 µM) application at the end of each measurement verified cell viability (inset on lower right of each panel). **C** cLTP strongly and persistently decreased [K^+^]_i_ in BK^+/+^ (n = 11 independent experiments from a total of n = 54 neurons obtained from N = 6 preparations). **D** cLTP induction failed to decrease [K^+^]_i_ in BK^−/−^ (n = 9 independent experiments with a total of n = 48 neurons obtained from N = 5 preparations). **E** BK inhibition by PAX (5 µM) prevented decrease of [K^+^]_i_ during cLTP (n = 10) independent experiments from a total of n = 43 neurons obtained from from N = 5 preparations). **F** Compared to vehicle, NMDAR inhibition by AP5 (100 µM) significantly reduced K^+^ efflux during cLTP (n = 9 independent experiments with a total of n = 37 neurons obtained from N = 5 preparations). **G** Compared to vehicle, LTCC inhibition by NIFE (5 µM) significantly reduced K^+^ efflux during cLTP (n = 6 independent experiments with a total of n = 52 neurons obtained from N = 4 preparations). **H** Maximum differences between average baseline and minimum YFP/CFP during cLTP recordings in panels C-G to corresponding BK^+/+^ condition: § = p ≤ 0.01; # = p ≤ 0.001. Statistics: One-way ANOVA with Dunnett's multiple comparison test. All bar diagrams presented as means ± SEM. See also Fig. S4 and Table S4

**Fig. 5:**
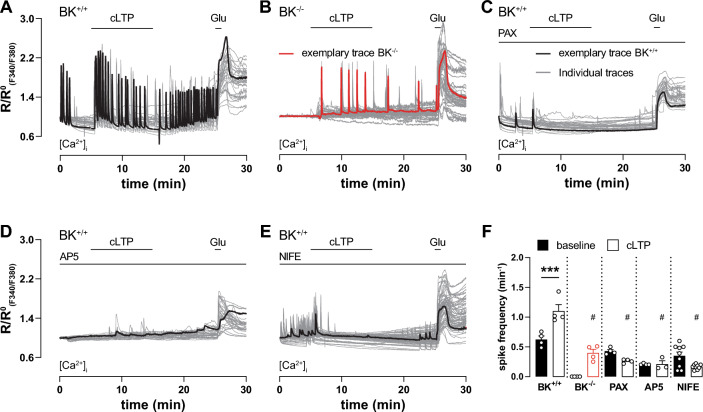
Ca^2+^ oscillations during cLTP induction depend on BK, NMDAR and LTCC. **A**–**E** Time course of ratio between fluorescence intensities at 340 nm and 380 nm (R_F340/F380_) in 9 DIV hippocampal neuronal cultures loaded with the Ca^2+^-sensitive dye Fura-2-AM. Traces recorded from individual neurons during multiple measurements are plotted in grey, representative traces in black except for (**B**) which is in red. Glutamate (20 µM) application at the end of each measurement verified cell viability and served as positive control. **A**, **B** Neuronal Ca^2+^ oscillations spontaneously occurred under control conditions in BK^+/+^ (**A**; n = 4 independent experiments with a total of n = 31 neurons obtained from 4 preparations) but not BK^−/−^ neurons (**B**; n = 4 independent experiments with a total of n = 35 neurons obtained from 3 preparations). In BK^+/+^, cLTP induction increased oscillation frequency to a significantly higher degree than in BK^−/−^. **C**–**E** Inhibition of BK, NMDAR, and LTCC by 10 min pre-incubation with 5 µM PAX (**C**; n = 4 independent experiments with a total of n = 29 neurons obtained from 3 preparations), 100 µM AP5 (**D**; n = 4 independent experiments with a total of n = 29 neurons obtained from 3 preparations) and 5 µM NIFE (**E**; n = 4 independent experiments with a total of n = 69 neurons obtained from 5 preparations), respectively, reduced spontaneous Ca^2+^ oscillations and prevented the cLTP-induced rise in frequency. **F** Spiking frequency before and after cLTP induction in indicated conditions. cLTP increased Ca^2+^ oscillation frequency in BK^+/+^ neurons. Genetically or pharmacological BK inhibition as well as AP5 and NIFE prevented frequency increase. Compared with corresponding BK^+/+^ condition: # = p ≤ 0.001. Statistics: Two-way ANOVA with Tukey's multiple comparison test. All bar diagrams presented as means ± SEM. See also Table S5

Statistical analyses were performed using GraphPad Prism 9.4.1. Data are expressed as mean ± standard error of mean (SEM). After data sets passed the Shapiro–Wilk test for Gaussian normal distribution, an unpaired (Figs. 1B; 2E and S3B, D) or paired (Figs. 2F, G) t-test was performed. Comparisons consisting of more than 2 data sets were tested with either one- or two-way ANOVA followed by a Dunnett’s (Fig. 4H), Sidak’s (Fig. 2A, B, F, G, 3; S1B-D and S2) or Tukey’s (Figs. 5F; S4K; S5C and S6F) multiple comparison test. The specific statistical tests used are explained in the respective legends. For an exact description, see also Tables S1–S5. P-values of ≤ 0.05 were represented with *, p ≤ 0.01 with **, and p ≤ 0.001 with ***, indicating comparisons between genotypes. Statistically significant differences to corresponding BK^+/+^ condition (Figs. 4H and 5F) were indicated by † = p ≤ 0.05; § = p ≤ 0.01; # = p ≤ 0.001.

## Results

### Characterization of conditional CA1-specific BK KO mice

Impaired MWM memory acquisition in BK^−/−^ was previously reported [28]. BK^−/−^ were also observed to suffer from cerebellar ataxia [30, 40], which could impact their MWM swimming performance. In order to determine whether memory performance is affected independently of motor performance, we generated a conditional KO line lacking the pore-forming BKα subunit in hippocampal CA1 pyramidal cells (cKO), the postsynaptic neurons of hippocampal Schaffer-collateral CA3 to CA1 synapses. Plasticity of these synapses is generally agreed to correlate with the animal’s performance in spatial learning tasks [41]. Ablation of the floxed BKα (BK^fl/+^) pore exon in CA1 pyramidal cells was achieved by intercrossing mice carrying the floxed allele to a transgenic subline (T29-1) expressing Cre recombinase under control of CaMKII (Cre^tg/+^) in CA1 pyramidal cells [29] to generate age- and litter-matched cKO (*genotype*: Cre^tg/+^; BK^fl/−^) and the corresponding controls (CTRL, *genotype*: Cre^tg/+^; BK^fl/+^). T29-1 was used in many studies to achieve recombination in CA1 pyramidal cells [42, 43]. It is one of several Cre-expressing sublines originally created by the Tonegawa lab under control of the CamKIIα promoter. All original founder lines display different expression patterns [29], with T29-1 causing very high recombination efficiency and also a remarkable selectivity in CA1 pyramidal cells. First, specific hippocampal recombination in cKO was confirmed by polymerase chain reaction (PCR) (Fig. S1A). Additionally, Western blots demonstrated significantly reduced immunoreactivity to a validated BKα antibody [44] in the hippocampus of cKO compared to CTRL (Fig. 1A, B) with tissue from wildtype (BK^+/+^) and BK^−/−^ serving as positive and negative controls, respectively. Consistent with the Cre expression pattern [29], BKα immunofluorescence was consistently reduced in CA1 but not CA3 or the dentate gyrus (DG) of cKO compared to CTRL as shown in hippocampal sections of adult mice. Again, absence of BKα immunoreactivity in slices from global BK^−/−^ confirmed antibody specificity (Fig. 1C). While Cre-mediated recombination is not completely restricted to CA1 pyramidal cells, cKO mice do not seem to express any BK in these cells. Therefore, these mice are well suited to test BK’s role in hippocampal synaptic plasticity and hippocampus-dependent learning paradigms [41].

### Anxiety behavior and motor abilities are normal in cKO

Comparison of learning behavior between genotypes in the MWM can be perturbed by differences in anxiety and motor abilities. Thus, open field and beam walk tests were performed to assess if cKO suffer from corresponding impediments. Both paradigms examine test subjects’ motor abilities [45, 46], while mouse behavior in the open field additionally represents an important indicator of anxiety levels [47, 48]. cKO and CTRL did not differ in anxiety and exploratory behavior as evidenced by similar amounts of time spent in the center of the open field arena (Fig. 1D) and similar number of rearings (Fig. 1E). During their time in the open field, animals from both genotypes did not differ in total distance travelled (Fig. 1F) and mean movement speed (Fig. 1G). These equivalent motor abilities of cKO and CTRL were further confirmed by the more complex beam walk test in which animals walk over round- and square-shaped beams of decreasing diameter and increasing difficulty. Neither falls or missteps nor the time to cross the beam differed between genotypes (Fig. 1H–J and Fig. S1B–D). Additionally, results from the beam walk test do not suggest any defects in the visual abilities of cKO. This is expected, as in T29.1 Cre activity is only sparsely detected in retinal bipolar, amacrine, and ganglion cells [49] and global BK knockout mice display unaltered electroretinography results under normal lighting conditions [50]*.* In summary we conclude that BKα is specifically depleted from hippocampal CA1 pyramidal cells of cKO mice, which do not display altered anxiety levels or locomotor abilities in comparison to their age- and litter-matched CTRLs.

### Hippocampal learning, memory and Schaffer collateral LTP is impaired in cKO

To test hippocampus-dependent spatial learning and cognitive performance, cKO and CTRL mice were subjected to a MWM task [51]. Over the course of five training days with four training sessions per day, mice learned to remember the hidden platform’s position in the NE quadrant. On the first day, CTRL and cKO exhibit similar latencies (Fig. 2A). On the second day already, performance in CTRL improved more quickly than in cKO. As evidenced by continuous shortening of the latency, cKO also learned the platform location over the course of 5 training days, but never reached the performance of CTRL (Fig. 2A). During probe trial on day 6, cKO latency to the platform location was similar to CTRL, but cKO showed significantly less target quadrant preference while spending significantly more time in the opposite SW quadrant than CTRL (Fig. 2B). The lack of target quadrant preference was evident when observing the animals’ swimming path. While CTRL focused on examining the target quadrant (Fig. 2C), cKO kept randomly scanning the arena (Fig. 2D). Swim speed as evaluated at the acquisition probe trial was similar between CTRL and cKO (Fig. 2E), ruling out swimming abilities as source for different latencies to reach the platform as well as quadrant preferences.

### Schaffer collateral LTP is defective in cKO

Acquisition of spatial memory in the MWM was repeatedly shown to correlate with NMDAR- dependent LTP of hippocampal Schaffer collateral synapses [41, 52]. Therefore, we measured hippocampal LTP by recording extracellular field potentials (fEPSP) in acute hippocampal slices. In accordance with MWM observations, HFS elicited stable LTP in CTRL, while initially potentiated fEPSP slopes dropped to baseline levels in cKO within 60 min (Fig. 2F**, **Table S2F). We could not find any indications that altered LTP is due to changes in basal synaptic transmission, as both genotypes displayed similar fEPSP amplitudes in response to a range of input strengths (Fig. S2D). Importantly, similar levels of paired-pulse facilitation (PPF) also indicated unaltered presynaptic function between CTRL and cKO (Fig. S2C).

Additionally, we also observed reduced LTP in a chemically induced LTP (cLTP) paradigm, in which synapses are not potentiated electrically, but chemically. We tested this key mechanism in cKO by applying cLTP, a model in which LTP is induced by application of 20 µM forskolin, to activate adenylyl cyclases, 0.1 µM rolipram to inhibit phosphodiesterase 4 (PDE4) and 50 µM picrotoxin, to block GABA_A_ receptors [32, 53, 54]. Interestingly, forskolin/rolipram/picrotoxin (FRP)-induced LTP was previously demonstrated to depend on NMDAR [32]. 10 min FRP perfusion of hippocampal slices sustainably increased initial fEPSP slopes in both genotypes to levels significantly higher than the respective baselines. This potentiation, however, was significantly less pronounced in cKO than CTRL (Fig. 2G, Table S2G). In both genotypes, FRP-induced comparatively more potentiation than HFS. This is probably the result of a more complete activation of the total neuronal population by the cLTP cocktail, while electrical stimulation is restricted to tissue directly adjacent to the stimulation electrode.

Hippocampal LTP is generally associated with MWM learning ability, while hippocampal LTD is linked to cognitive flexibility as observed during MWM reversal training [55, 56]. Recently, defective hippocampal LTD and an associated reduction in reversal learning and cognitive flexibility were described in mice lacking the Na^+^-activated K^+^ channel Slack (Sequence Like a Ca^2+^-activated K^+^ Channel, K_Na_1.1, Slo2.2) which is highly homologous to BK [31, 38]. We therefore conducted reversal training subsequent to the acquisition phase of the MWM by placing the platform on the opposite SW side and by mirroring the animals' insertion points. As compared to the last acquisition trial(s) and the acquisition probe trials, both genotypes took longer to locate the new platform position in the reversal phase (Fig. S2A), but latencies were comparable between CTRL and cKO throughout of the reversal phase. Apparently, both cKO and CTRL were able to effectively update their memory of the new platform location (Fig. S2B), indicating comparable memory flexibility in both genotypes.

Together, our behavioral and electrophysiological data suggest that BK is necessary for normal hippocampal synaptic plasticity in the form of MWM learning and Schaffer collateral LTP.

### BK deletion does not alter glutamate receptor subunit composition

Function of heterotetrameric NMDA- [57] and AMPA- [21] receptor complexes necessary for synaptic transmission and plasticity is crucially determined by their respective subunit composition. It was previously reported that animals lacking the closely related Na^+^-activated K^+^ channel Slack show decreased hippocampal GluN2B expression and impaired hippocampal synaptic plasticity [38]. We therefore examined protein levels of the most prevalent hippocampal NMDAR and AMPAR subunits [58, 59] in adult cKO and CTRL prior to or after completion of the MWM task. Expression levels were not different between cKO and CTRL for GluN1, GluN2A, GluN2B, GluA1 and GluA2 in the naïve state (Fig. S3A) nor for GluN2A, GluN2B and GluA1 after completion of the MWM task (Fig. S3B). GluN1 and GluA2 could not be analyzed due to low sample availability. Taken together, this suggests that defective hippocampal learning, memory and LTP in cKO is not due to altered glutamate receptor subunit expression.

### Reduced GluA1-S845 phosphorylation in cKO after LTP induction

Induction of LTP by NMDAR stimulation initiates a signaling cascade resulting in GluA1 phosphorylation at S845, which is a crucial step to allow LTP expression by regulated AMPAR exocytosis [60–62]. We used cLTP to test this key mechanism in cKO. Compared to vehicle-treated controls, cLTP induction for 10 min with FRP (20 µM/0.1 µM/50 µM) significantly increased S845 phosphorylation in hippocampal slices of CTRL but not cKO (Fig. 3A). As shown earlier [54], cLTP induction by FRP did not influence GluA1 S831 phosphorylation confirming specificity of the elicited signaling (Fig. 3B). Interestingly, however, acute pharmacological blockade of BK channels with paxilline (PAX; 5 µM) effectively prevented cLTP-induced GluA1 S845 phosphorylation in CTRL, while PAX was less effective in cKO (Fig. 3C). In both genotypes, pharmacological BK blockade did not influence GluA1 S831 phosphorylation levels (Fig. 3D). This confirms our electrophysiological and MWM studies that suggest a pivotal role for BK in the cellular signaling mechanisms necessary for the expression of hippocampal LTP as well as hippocampus-dependent spatial learning and memory.

### BK-mediated K^+^ efflux sustains neuronal Ca^2+^ oscillations during cLTP

To examine in detail how BK supports hippocampal LTP and, in turn, hippocampus-dependent memory formation, we next performed live cell imaging of dissociated hippocampal neuronal cultures generated from homozygous BK^+/+^ and BK^−/−^ pups. Neurons were virally transduced with the genetically encoded potassium ion indicator (GEPII) NES lc-lysM GEPII 1.0 [35, 36] (Fig. 4A) or loaded with the Ca^2+^-sensitive dye Fura-2-AM (Fig. 5). For NES lc-LysM GEPII 1.0, the ratio of Förster resonance energy transfer (FRET) fluorescence divided by cyan fluorescent protein (CFP) fluorescence is directly proportional to [K^+^]_i_. GEPII sensitivity to [K^+^]_i_ changes was verified by reproducible rapid decrease of the FRET/CFP ratio after repeated perfusion of 20 µM glutamate for 10 s presumably in response to neuronal depolarization (Fig. 4B). Additionally, we observed antiparallel progression of single fluorescent protein fluorescence intensity traces recorded over time, which indicates a valid FRET signal (Figs. 4B and S4A–E). cLTP induction by FRP application massively reduced [K^+^]_i_ in BK^+/+^ (Fig. 4C and H). This drop in [K^+^]_i_ was not observed after cLTP induction in BK^−/−^ neurons (Fig. 4D and H) or after BK inhibition by PAX in BK^+/+^ (Fig. 4E and H). Neuron viability and physiological functionality was verified by glutamate application after each measurement (insets in Fig. 4C–G). This suggests that BK channels are necessary for sustained [K^+^]_i_ depression during and after cLTP induction. Surprisingly, inhibition of both, NMDAR (Fig. 4F) as well as LTCC (Fig. 4G) prevented [K^+^]_i_ reduction during cLTP by about 50% (Fig. 4H). This indicates that K^+^ outflow during cLTP depends on LTCC and NMDAR as sources for the high local [Ca^2+^]_i_ required for BK activation. Comparison of the mean baseline between non-normalized curves of all K^+^ sensitive measurements (Fig. S4F–J) revealed a comparable basal [K^+^]_i_ in both genotypes (Fig. S4K).

The sustained reduction of [K^+^]_i_ after cLTP induction (Fig. 4C) was surprising. To evaluate whether it might affect the neuronal resting membrane potential, we elicited cLTP in the presence of the membrane potential-sensitive dye DiBAC_4_(3) [39]. The dye enters depolarized cells leading to increased fluorescence. Upon cLTP induction by FRP, DiBAC_4_(3) fluorescence was not significantly affected in either BK^+/+^ and PAX-inhibited BK^+/+^, while glutamate application provoked a rapid and profound increase in DiBAC_4_(3) fluorescence (Fig. S5A–C). The fact that the membrane potential hardly changes despite a sustained K^+^ efflux (Fig. 4C) might be a consequence of sustained Ca^2+^ influx through Ca^2+^ oscillations during cLTP and washout (Fig. 5A), which could offset the increased K^+^ permeability to result in a constant net membrane potential.

### BK supports Ca^2+^ oscillations during LTP

Although high local [Ca^2+^]_i_ is necessary for BK activation, BK channel activity also supports Ca^2+^ entry by rapidly repolarizing the plasma membrane to return voltage-gated Na^+^ or Ca^2+^ channels into an activatable state to support fast AP firing rates [14–16]. To test this reciprocal relationship, we induced cLTP in hippocampal neurons loaded with the Ca^2+^-sensitive dye Fura-2-AM (Fig. 5). In line with previous observations [63], spontaneous Ca^2+^ spikes were observed under basal conditions in 86% of all BK^+/+^ neurons (Fig. 5A). In all BK^+/+^ neurons, however, cLTP led to a significant increase in Ca^2+^ oscillation frequency that was preserved after wash-out (Fig. 5A and F). Interestingly, no spontaneous basal activity was observed in neurons lacking BK (Fig. 5B) while spiking activity in these cells was also increased by cLTP albeit at a significantly lower frequency than in BK^+/+^ (Fig. 5F). When BK activity was pharmacologically blocked in BK^+/+^, basal Ca^2+^ oscillations were only observed in 45% of these neurons and their frequency did not increase during or after cLTP induction (Fig. 5C and F). As expected, blocking neuronal Ca^2+^ entry through NMDAR and LTCC, by AP5 and NIFE, respectively, prevented FRP cLTP-induced increases in Ca^2+^ spiking frequency (Fig. 5D–F). Spontaneous activity was observed under NIFE, but not AP5. Comparison of mean baselines from non-normalized curves of all Ca^2+^ sensitive measurements (Fig. S6A–E) indicates equivalent basal [Ca^2+^]_i_ in both genotypes (Fig. S6F).

In conclusion, cLTP-provoked Ca^2+^ spikes require NMDAR and LTCC in addition to functional BK expression. Hence, the low-frequency activity of hippocampal neurons lacking BK lays basis for the impaired expression of hippocampal LTP and deficits in spatial learning and memory performances in cKO.

## Discussion

Here we report efficient hippocampus-specific conditional BK deletion (cKO. Figs. 1A–C and S1A) by Cre-mediated recombination under control of the CA1-selective T29-1 Cre subline [29]. Impairment of spatial learning and memory performance in the MWM as well as reduced electrically and chemically induced hippocampal LTP in cKO (Fig. 2) confirms an earlier report of restricted MWM learning in BK^−/−^ global BK KO mice [28]. Unlike BK^−/−^, however, cKO do not display motor deficits like tremor and ataxia (Fig. 1D–J and S1B–D) previously reported to affect BK^−/−^ [30, 40] that might interfere with their performance in behavioral assays.

LTP expression relies on activation of postsynaptic signaling mechanisms by Ca^2+^ entry through NMDAR and LTCC [64]. This increased postsynaptic [Ca^2+^]_i_, in turn, promotes insertion of additional AMPAR into postsynaptic sites after phosphorylation of the GluA1 subunit at either S831 or S845 to ultimately enhance synaptic transmission efficiency [19–21, 52]. In accordance with lack of hippocampal LTP, cLTP induction in cKO failed to increase phosphorylation levels at S845 of the AMPAR subunit GluA1, while phosphorylation at S831 remained unaffected (Fig. 3A, B). Additionally, S845 phosphorylation during cLTP was sensitive to acute BK inhibition by PAX (Fig. 3C). This suggests that BK activity during LTP induction is necessary for LTP expression through GluA1 phosphorylation at S845 that, in turn, increases AMPAR conductance and exocytosis of AMPAR from intracellular compartments [21]. BK might affect protein kinase A (PKA)-mediated S845 phosphorylation by supporting postsynaptic Ca^2+^ entry through NMDAR or LTCC to activate Ca^2+^-dependent adenylyl cyclase [65]. Alternative explanations for impaired hippocampal LTP were ruled out by confirming normal synaptic function (Fig. S2C, D) as well as AMPAR and NMDAR subunit composition in cKO (Fig. S3). Interestingly, cKO performed equal to CTRL in the reversal phase of the MWM (Fig. S2A), which tests the memory flexibility of the experimental animals. Many studies suggest that physiological LTD may be the base for proper memory flexibility [55, 56]. This stands in contrast to adult Slack KO, which are deficient in MWM reversal learning [31] and display normal LTP [38]. Thus, even as BK and Slack share high sequence homology and several topological features, both channels seem to serve very different functions in hippocampal synaptic plasticity. In addition to BK and Slack, small-conductance Ca^2+^-activated K^+^ channels (SK), which are also gated by Ca^2+^ to cause an efflux of K^+^ yielding a single channel conductance of 10–20 pS, are widely expressed in central neurons including the postsynaptic membrane of glutamatergic synapses [66]. Blocking SK channels with apamin promoted hippocampal synaptic plasticity and improved spatial learning in the MWM task by reducing SK-mediated AHP to disinhibit NMDAR [67–69]. In contrast to post-synaptic BK function, these findings suggest that the repolarizing conductance provided by SK activation decreases hippocampal glutamate receptor-mediated depolarization and thereby synaptic plasticity, neuronal excitability, and memory formation [66, 70, 71]. However, apamin-sensitive SK channels are also present at intracellular sites, and they are widely distributed throughout the surface of hippocampal neurons as well as in interneurons. Thus, multiple SK channel pools may indirectly contribute to LTP expression and thereby to learning performance [72–74].

Patients carrying both gain- and loss-of-function (LOF) mutations of BK suffer from multifaceted combinations of movement and seizure disorders as well as developmental delay and intellectual disability [1]. Cognitive development and neuronal functions like learning and memory are associated with synaptic plasticity [17, 18]. Thus, our finding of impaired hippocampal LTP might explain cognitive impairment in these patients, particularly in the case of LOF mutations. Additionally interesting in this context is that reduced BK expression was associated with Alzheimer's disease (AD), while reduced LTP was demonstrated in synapses from AD patients and related mouse models [75, 76]. Accordingly, pharmacological BK activation not only rescues hippocampal LTP in a mouse model of AD but also recovers cognitive function as assessed by behavioral assays [76].

How could BK influence hippocampal synaptic plasticity in the form of LTP? LTP induction depends on postsynaptic Ca^2+^ entry. Accordingly, we observe that the frequency of spontaneous Ca^2+^ oscillations in dissociated hippocampal neuronal cultures was greatly raised by cLTP induced signaling (Fig. 5A). Concomitant to this increased Ca^2+^ spike activity, we observed a massive BK-dependent reduction in [K^+^]_i_ (Fig. 4C). We assume that this outflow of K^+^ through BK is triggered by Ca^2+^ entry through NMDAR and LTCC (Fig. 4F and G). Both of these channels were previously demonstrated to physically interact with postsynaptic BK channels [7, 8] and to provide elevated local [Ca^2+^]_i_ necessary for BK activation [7, 9–11]. It is therefore tempting to speculate that BK activation is one of the mechanisms by which both NMDAR and LTCC exert their essential contribution to the expression of hippocampal LTP [19, 20]. In contrast, inhibition of each NMDAR and LTCC substantially reduced Ca^2+^ during cLTP (Fig. 5D and E), while the reduction of [K^+^]_i_ was still at about half the amplitude of the control (Fig. 4F–H). It is conceivable that this drop in [K^+^]_i_ is due to Ca^2+^-independent BK activation e.g. by direct phosphorylation of BK [77] or due to altered assembly with auxiliary subunits [78]. Alternatively, each Ca^2+^ source (NMDAR and LTCC) might activate a locally associated BK population. This would slightly reduce cellular [K^+^]_i_, but not sufficiently to support LTP. Only the simultaneous activity of both Ca^2+^ sources raises global [Ca^2+^]_i_ sufficiently to trigger LTP and to efficiently activate BK channels.

While Ca^2+^ entry activates BK channels, we were surprised to discover that BK activity, in contrast to SK activity [70, 79], seems to support neuronal Ca^2+^ oscillations (Fig. 4C and D). This seems contradictory, as neuronal hyperpolarization by K^+^ channels in general and BK in particular is thought to inhibit NMDAR as well as voltage-gated cation channels [8, 80]. It has, however, also been proposed that BK is involved in fast repolarization after APs [14–16]. Consistent with our observations, BK inhibition was previously shown to slow the firing rate of hippocampal CA1 pyramidal cells, GABAergic neurons, and intracardiac autonomic neurons [15, 81, 82]. Others, however, reported increased firing frequencies after BK blockade in CA1 pyramidal neurons of the rat [83].

So far, we assumed neurons to act as single functional units despite their well-known morphological subdivision into different compartments. An increasing body of evidence, however, suggests specialized functions of dendritic compartments, particularly in respect to synaptic plasticity [84–87]. Fear learning-induced increases in local Ca^2+^, for example, were found in apical but not basal dendrites of layer 2/3 (L2/3) pyramidal neurons in the mouse auditory cortex [88]. Paradoxically, just in those apical dendrites an inactivating function of BK could be observed [89], which consequently leads to decrease in neuronal excitation as BK is activated by Ca^2+^ spikes mediated by NMDAR [90] and voltage-gated Ca^2+^ channels [91]. This contrasts with our findings suggesting positive feedback involvement of somatic BK in LTP through fAHP (Figs. 4C and 5A). While this seems like a contradiction at first glance, dendritic BK may nevertheless support increased plasticity through three factors. First, by hyperpolarizing the membrane potential, BK could impede the triggering of action potentials by synaptic inputs [89]. This could increase the specificity of synaptic potentiation, as only closely tuned inputs can cross the increased threshold [92]. Next, excessive and thus neurotoxic Ca^2+^ accumulation may be prevented, so that fewer synapses or dendrites perish [12]. Lastly, hyperpolarization in apical dendrites by BK may influence the timing of postsynaptic potentials and Ca^2+^ spikes to optimize temporal coding and signal summation, which might facilitate coincidence detection and thus also the occurrence of LTP [93].

Based on the presented data we propose a mechanism by which BK is activated by Ca^2+^ entry through both NMDAR and LTCC in postsynaptic membranes. BK-mediated K^+^ outflow, in turn, amplifies Ca^2+^ spiking frequency, which facilitates Ca^2+^ entry in support of the Ca^2+^-mediated mechanisms ultimately leading to AMPAR phosphorylation and LTP expression.

### Supplementary Information

Below is the link to the electronic supplementary material.

Supplementary file1 (PDF 1470 KB)

## Data Availability

All data generated or analyzed during this study that are not included in this published article and its supplementary information files are available from the corresponding authors on reasonable request. Further information and requests for resources and reagents should be directed to and will be fulfilled either by the Lead Contact, Lucas Matt (lucas.matt@uni-tuebingen.de) or by Robert Lukowski (robert.lukowski@uni-tuebingen.de). This study includes no data deposited in external repositories.
