# Supplementary material for: BK channels sustain neuronal Ca2+ oscillations to support hippocampal long-term potentiation and memory formation
Source: Cell Mol Life Sci. 2023 Nov 21;80(12):369. doi: 10.1007/s00018-023-05016-y (PMC10663188; doi:10.1007/s00018-023-05016-y)
Supplement: Supplementary file 1 — Supplementary file1 (PDF 1470 KB) [file 18_2023_5016_MOESM1_ESM.pdf]

Supplementary Materials for the manuscript

BK channels sustain neuronal Ca^{2+} oscillations to support hippocampal long-term potentiation and memory formation in mice

Thomas Pham[1], Tamara Hussein[1], Dila Calis[2], Helmut Bischof[1], David Skrabak[1], Melanie Cruz Santos[1], Selina Maier[1], David Spähn[1], Daniel Kalina[1], Stefanie Simonsig[1], Rebekka Ehinger[1], Bernhard Groschup[3], Marlies Knipper[2], Nikolaus Plesnila[3,4], Peter Ruth[1], Robert Lukowski[1,§], Lucas Matt[1,5,§]

[1]Department of Pharmacology, Toxicology and Clinical Pharmacy, Institute of Pharmacy, University of Tübingen, Tübingen, Germany
[2]Department of Otolaryngology, Head and Neck Surgery, Molecular Physiology of Hearing, Tübingen Hearing Research Centre, University of Tübingen, Tübingen, Germany
[3]Laboratory of Experimental Stroke Research, Institute for Stroke and Dementia Research (ISD), University Hospital, Ludwig-Maximilians-University Munich (LMU), Munich, Germany
[4]Munich Cluster for Systems Neurology (SyNergy), Munich, Germany
[5]Lead contact
[§]These authors equally contributed to the study

Correspondence: Lucas Matt, email: lucas.matt@uni-tuebingen.de.

SUPPLEMENTAL TABLES

Table S1. Values and statistics for Figure 1.

Panel						
**B**	**genotype**	**relative expression**		**n**	**statistics**	**p**
	CTRL	0.258 ± 0.015		3	unpaired t-test	
	cKO	0.200 ± 0.014		3		0.045 vs. CTRL
**D**	**genotype**	**time in center (%)**		**n**	**statistics**	**p**
	CTRL	17.49 ± 2.42		14	unpaired t-test	
	cKO	18.37 ± 1.35		14		0.75 vs. CTRL
**E**	**genotype**	**rearings**		**n**	**statistics**	**p**
	CTRL		51.36 ± 7.780	14	unpaired t-test	
	cKO		44.07 ± 3.427	15		0.39 vs. CTRL
**F**	**genotype**	**total distance (m)**		**n**	**statistics**	**p**
	CTRL		173.0 ± 12.02	14	unpaired t-test	
	cKO		198.9 ± 12.43	15		0.15 vs. CTRL
**G**	**genotype**	**mean speed (cm/s)**		**n**	**statistics**	**p**
	CTRL		9.614 ± 0.668	14	unpaired t-test	
	cKO		11.10 ± 0.672	15		0.13 vs. CTRL
**H**	**genotype**	**beam**	**falls per trial**	**n**	**statistics**	**p**
	CTRL	square	0.144 ± 0.038	15	$F_{1,354}$ = 0.14<br>p = 0.70<br><br>2-way ANOVA with Sidak's multiple comparison	
		round	0.100 ± 0.051	15		
		square & round	0.122 ± 0.030	15		
	cKO	square	0.144 ± 0.048	15		0.98 vs. CTRL
		round	0.100 ± 0.048	15		0.84 vs. CTRL
		square & round	0.122 ± 0.032	15		0.997 vs. CTRL
**I**	**genotype**	**beam**	**slips per trial**	**n**	**statistics**	**p**
	CTRL	square	0.281 ± 0.038	12	$F_{1,180}$ = 0.18<br>p = 0.68<br><br>2-way ANOVA with Sidak's multiple comparison	
		round	0.306 ± 0.082	12		
		square & round	0.302 ± 0.046	12		
	cKO	square	0.539 ± 0.082	14		0.0092 vs. CTRL
		round	0.147 ± 0.041	13		0.19 vs. CTRL
		square & round	0.353 ± 0.047	14		0.91 vs. CTRL
**J**	**genotype**	**beam**	**latency per trial**	**n**	**statistics**	**p**
	CTRL	square	12.321 ± 1.517	14	$F_{1,290}$ = 2.88<br>p = 0.09<br><br>2-way ANOVA with Sidak's multiple comparison	
		round	9.601 ± 1.008	13		
		square & round	11.073 ± 0.937	14		
	cKO	square	10.826 ± 1.751	14		0.77 vs. CTRL
		round	8.631 ± 0.795	13		0.93 vs. CTRL
		square & round	9.690 ± 0.947	14		0.81 vs. CTRL

Table S2. Values and statistics for Figure 2.

Panel	genotype	days of training	latency (s)	n	statistics	p
A	CTRL	1	52.408 ± 1.795	20	$F_{1,234}$ = 16.74<br>p < 0.001<br>2-way ANOVA with Sidak's multiple comparison	
	cKO		54.171 ± 2.032	21		0.0017 vs. CTRL
	CTRL	2	21.833 ± 2.246	20		
	cKO		30.868 ± 3.281	21		0.03 vs. CTRL
	CTRL	3	16.804 ± 2.025	20		
	cKO		23.382 ± 3.116	21		0.09 vs. CTRL
	CTRL	4	11.351 ± 1.244	20		
	cKO		18.178 ± 2.522	21		0.02 vs. CTRL
	CTRL	5	9.019 ± 0.651	20		
	cKO		13.352 ± 1.831	21		0.03 vs. CTRL
	CTRL	Probe	9.919 ± 1.124	20		
	cKO		12.846 ± 2.796	21		0.57 vs. CTRL

Panel	genotype	quadrant	latency (s)	n	statistics	p
B	CTRL	NE	38.907 ± 2.630	20	$F_{1,156}$ = 0.03<br>p = 0.85<br>2-way ANOVA with Sidak's multiple comparison	
	cKO		30.825 ± 1.875	21		0.047 vs. CTRL
	CTRL	NW	19.754 ± 1.617	20		
	cKO		20.841 ± 1.701	21		
	CTRL	SW	15.864 ± 1.527	20		0.03 vs. CTRL
	cKO		22.389 ± 1.669	21		
	CTRL	SE	21.411 ± 2.294	20		
	cKO		22.879 ± 1.827	21		

Panel	genotype	swim speed (cm/s)	n	statistics	p
E	CTRL	22.27 ± 0.52	20	**unpaired t-test**	
	cKO	22.53 ± 0.59	21		0.65 vs. CTRL

Panel	genotype	baseline (%-baseline)	stimulated (%-baseline)	n (slice/ animal)	statistics	p
F	CTRL	100.8 ± 0.40	138.61 ± 8.21	7 / 4	$F_{1,195}$ = 83.31<br>p < 0.0001<br>2-way ANOVA	0.0037 vs. Baseline (paired t-test)
	cKO	99.30 ± 0.78	109.90 ± 8.00	8 / 4		0.1986 vs. Baseline (paired t-test)<br>0.0267 vs. stimulated CTRL (unpaired t-test)

Panel	genotype	baseline (%-baseline)	stimulated (%-baseline)	n (slice/ animal)	statistics	p
G	CTRL	101.1 ± 0.75	207.3 ± 31.86	10 / 4	$F_{1,276}$ = 72.41<br>p < 0.0001<br>2-way ANOVA	0.0089 vs. Baseline (paired t-test)
	cKO	104.7 ± 1.18	134.5 ± 8.86	10 / 5		0.0084 vs. Baseline (paired t-test)<br>0.0410 vs. stimulated CTRL (unpaired t-test)

Table S3. Values and statistics for Figure 3.

Panel	genotype	stimulated / unstimulated	pS845 / GluA1	n	statistics	p
A	CTRL	unstimulated	1.000 ± 0.223	4	$F_{1,11} = 31.85$ $p < 0.0002$ 2-way ANOVA with Sidak's multiple comparison	
	cKO		1.250 ± 0.141	4		
	CTRL	stimulated	4.376 ± 0.772	3		0.0006 vs. unstim. CTRL
	cKO		2.216 ± 0.365	4		0.02 vs. stim. CTRL

Panel	genotype	stimulated / unstimulated	pS831 / GluA1	n	statistics	p
B	CTRL	unstimulated	1.216 ± 0.170	4	$F_{1,12} = 0.002$ $p = 0.97$ 2-way ANOVA with Sidak's multiple comparison	
	cKO		1.000 ± 0.344	4		
	CTRL	stimulated	1.120 ± 0.363	4		>0.99 vs. unstim. CTRL
	cKO		1.069 ± 0.334	4		>0.9999 vs. stim. CTRL

Panel	genotype	stimulated / unstimulated	pS845 / GluA1	n	statistics	p
C	CTRL x PAX	unstimulated	1.000 ± 0.294	3	$F_{1,8} = 0.17$ $p = 0.69$ 2-way ANOVA with Sidak's multiple comparison	
	cKO x PAX		0.848 ± 0.215	3		
	CTRL x PAX	stimulated	0.827 ± 0.220	3		0.9972 vs. unstim. CTRL
	cKO x PAX		1.218 ± 0.225	3		0.8649 vs. stim. CTRL

Panel	genotype	stimulated / unstimulated	pS831 / GluA1	n	statistics	p
D	CTRL x PAX	unstimulated	1.000 ± 0.700	3	$F_{1,8} = 0.04$ $p = 0.86$ 2-way ANOVA with Sidak's multiple comparison	
	cKO x PAX		0.962 ± 0.803	3		
	CTRL x PAX	stimulated	0.766 ± 0.452	3		>0.9999 vs. unstim. CTRL
	cKO x PAX		0.953 ± 0.581	3		>0.9999 vs. stim. CTRL

Table S4. Values and statistics for Figure 4.

Panel	condition	$R/R^0_{FRET/CFP}$	n	statistics	p
H	$BK^{+/+}$	0.161 ± 0.021	11	$F_{4,42} = 4.28$	
	$BK^{-/-}$	0.034 ± 0.005	11	$p < 0.005$	<0.001 vs. $BK^{+/+}$
	PAX	0.019 ± 0.005	10		<0.001 $BK^{+/+}$
	AP5	0.080 ± 0.010	9	2-way ANOVA with Dunnett's multiple comparison	<0.001 vs. $BK^{+/+}$
	NIFE	0.090 ± 0.008	6		0.002 vs. $BK^{+/+}$

Table S5. Values and statistics for Figure 5.

Panel						
**F**	**stimulated / unstimulated**	**cond.**	**$R/R^0_{FRET/CFP}$**	**n**	**statistics**	**p**
	unstimulated	CTRL	0.6232 ± 0.059	4	$F_{5,23} = 2.52$<br>$p = 0.06$<br>1-way ANOVA with Tukey's multiple comparison	
		KO	0 ± 0	4		
		PAX	0.4278 ± 0.027	4		
		AP5	0.2071 ± 0.007	4		
		NIFE	0.3498 ± 0.062	9		
	stimulated	CTRL	1.099 ± 0.111	4	$F_{9,39} = 2.71$<br>$p < 0.01$<br>1-way ANOVA with Tukey's multiple comparison	<0.0001 vs. CTRL baseline
		KO	0.3967 ± 0.063	4		<0.0001 vs. CTRL cLTP
		PAX	0.2603 ± 0.016	4		<0.0001 vs. CTRL cLTP
		AP5	0.2089 ± 0.058	3		<0.0001 vs. CTRL cLTP
		NIFE	0.1659 ± 0.014	9		<0.0001 vs. CTRL cLTP

Table S6. Oligonucleotides used in this study.

genotyping

	primer	sequence 5' to 3'
**BK**	for1	TGG TCT TCT TCA TCC TCG GG
	for2	AAG GGC CAT TTT GAA GAC GTC
	rev	CCA GCC ACG TGT TTG TTG G
**T29.1-Cre**	for	CGT CCA TCT GGT CAG AAA AG
	rev	TCT TCT TCT TGG GCA TGG TC

Table S7. Primary antibody information.

Target	Source	Catalog #	Species / Isotype	Figure
BKα	NeuroMab	75-022	mouse IgG2a	1
GluA1	Origene	TA326534	mouse IgG1	3
$GluA1^{pS845}$	Millipore	AB5849	rabbit polyclonal	3
$GluA1^{pS831}$	Cell Signaling	75574	rabbit polyclonal	3
GluA2	Cell Signaling	13607	rabbit polyclonal	S3
GluN1	Origene	TA326536	mouse IgG1	S3
GluN2A	Origene	TA326537	mouse IgG2a	S3
GluN2B	Cell Signaling	14544	rabbit polyclonal	S3
α-Tubulin	Cell Signaling	3873	mouse IgG1	1, 3, S3

SUPPLEMENTAL FIGURES AND LEGENDS

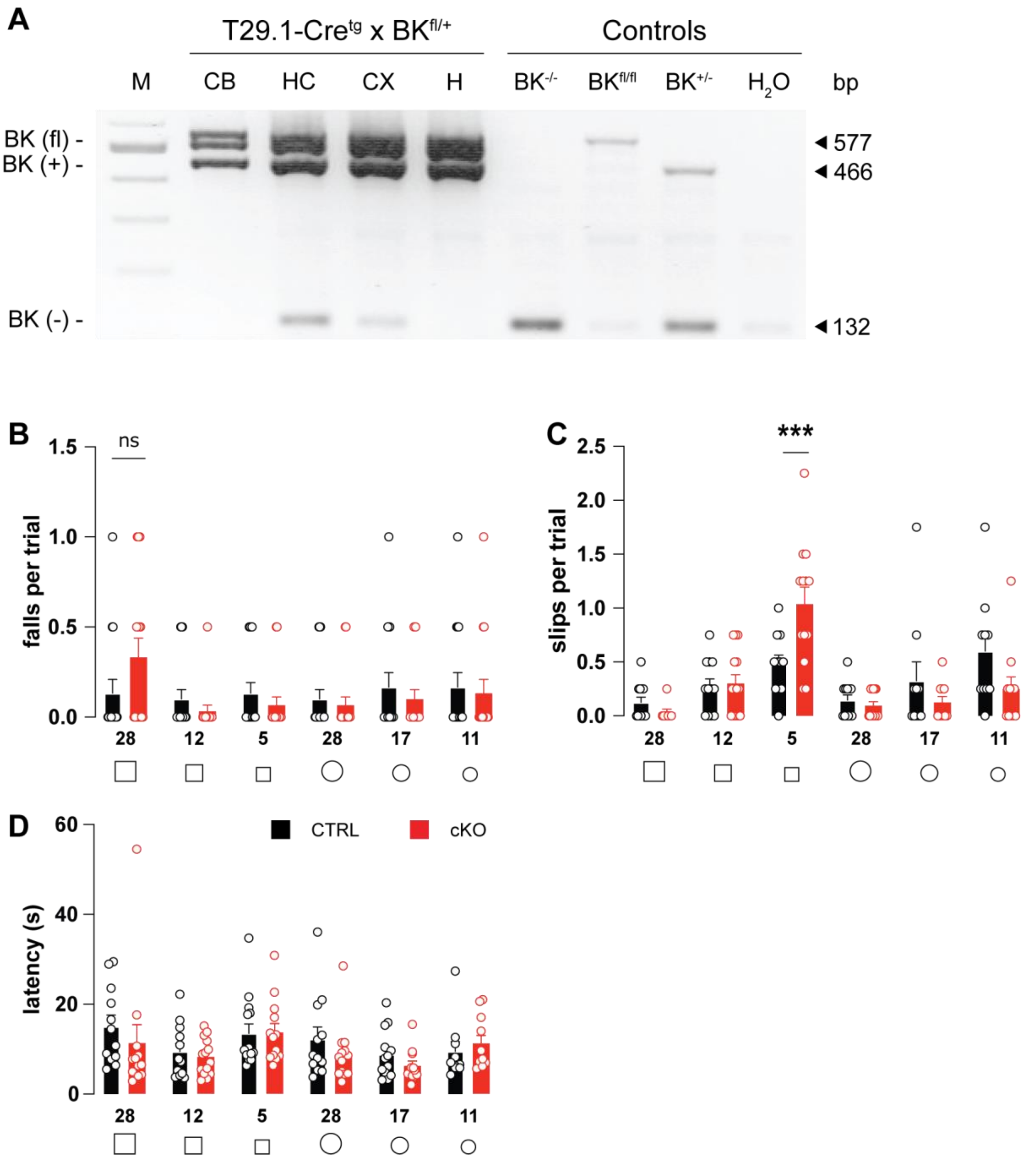

Figure S1. *Conditional Cre recombination and beam walk test in cKO*

(**A**) DNA isolated from indicated tissues of T29.1-Cre$^{tg/+}$; BK$^{fl/+}$ was amplified by genotyping PCR and separated by agarose gel electrophoresis. As expected, the wildtype allele (+) was detected at 466 bp. Recombination of the floxed allele ([fl], 577 bp) into the recombined null allele ([-], 132 bp) is prominently observed in hippocampus (HC) and faintly in cortex (CX). No recombination is observed in cerebellum (CB) and heart (H). Tissue from BK$^{-/-}$, BK$^{fl/fl}$, BK$^{+/-}$ as

well as water (H_2O) served as internal controls. n = 3 independent experiments from N = 3 animals.

(**B-D**) Results of the beam walk test for indicated individual beam shapes and sizes. (**B**) Falls per trial (N = 15 animals), (**C**) slips per trial (N = 8-14) and (**D**) latency to cross the beam (N = 11-14) did not differ between CTRL and cKO. An exception was the 5 mm square beam on which one individual subject had difficulties during its performance. N = 8-14. Statistics: Two-way ANOVA with Sidak's multiple comparison test. All bar diagrams presented as means ± SEM.

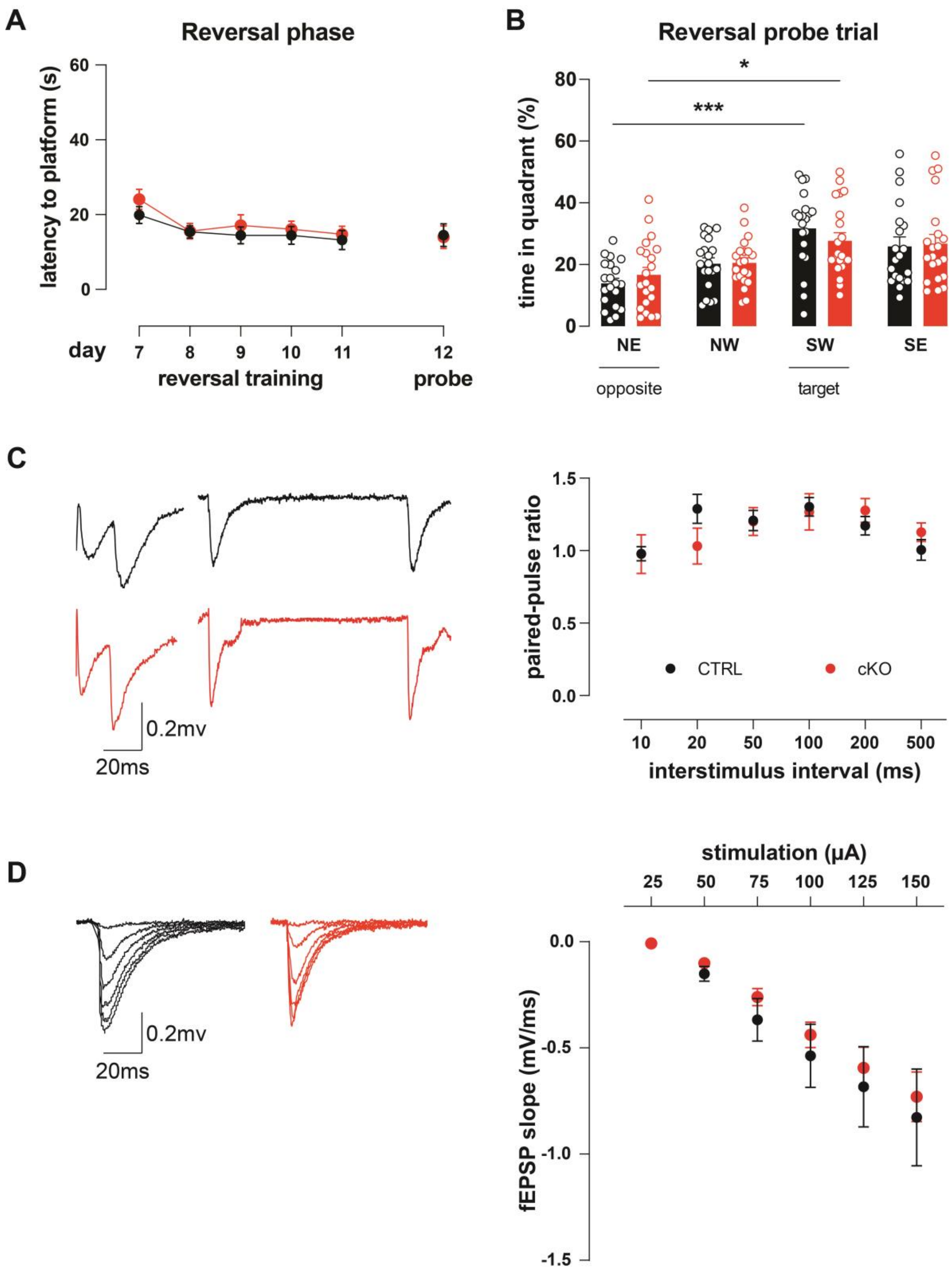

Figure S2. *Normal reversal learning and basal synaptic transmission in cKO*

(**A-B**) cKO display normal reversal learning in the MWM.

(**A**) Mean latencies to reach newly positioned hidden platform on 5 reversal training days (4 training sessions per day) and the reversal probe trial were not different between cKO

(N = 21 animals) and CTRL (N = 20). Since the hidden platform was removed, latency during probe trial on day 12 resulted from reaching the previous platform position. The latency of the probe trial is visually offset as it is not part of the training phase.

(**B**) Neither CTRL nor cKO showed significant quadrant preferences during reversal probe trial.

(**C-D**) Schaffer-collateral fEPSP initial slopes recorded from forebrain slices.

(**C**) Averaged PPF was not different between CTRL (n = 6 slices from N = 4 animals) and cKO (n = 6 slices from N = 4 animals) for all inter-stimulus intervals. Representative traces from CTRL (black) and cKO (red) shown left.

(**D**) Averaged initial fEPSP slopes recorded at stimulation intensities of 25 – 150 µA in 25 µA increments were not different between cKO (n = 8 slices from N = 5 animals) and CTRL (n = 8 slices from N = 5 animals). Left: Representative traces.

Statistics: Two-way ANOVA with Sidak's multiple comparison test. All bar diagrams presented as means ± SEM.

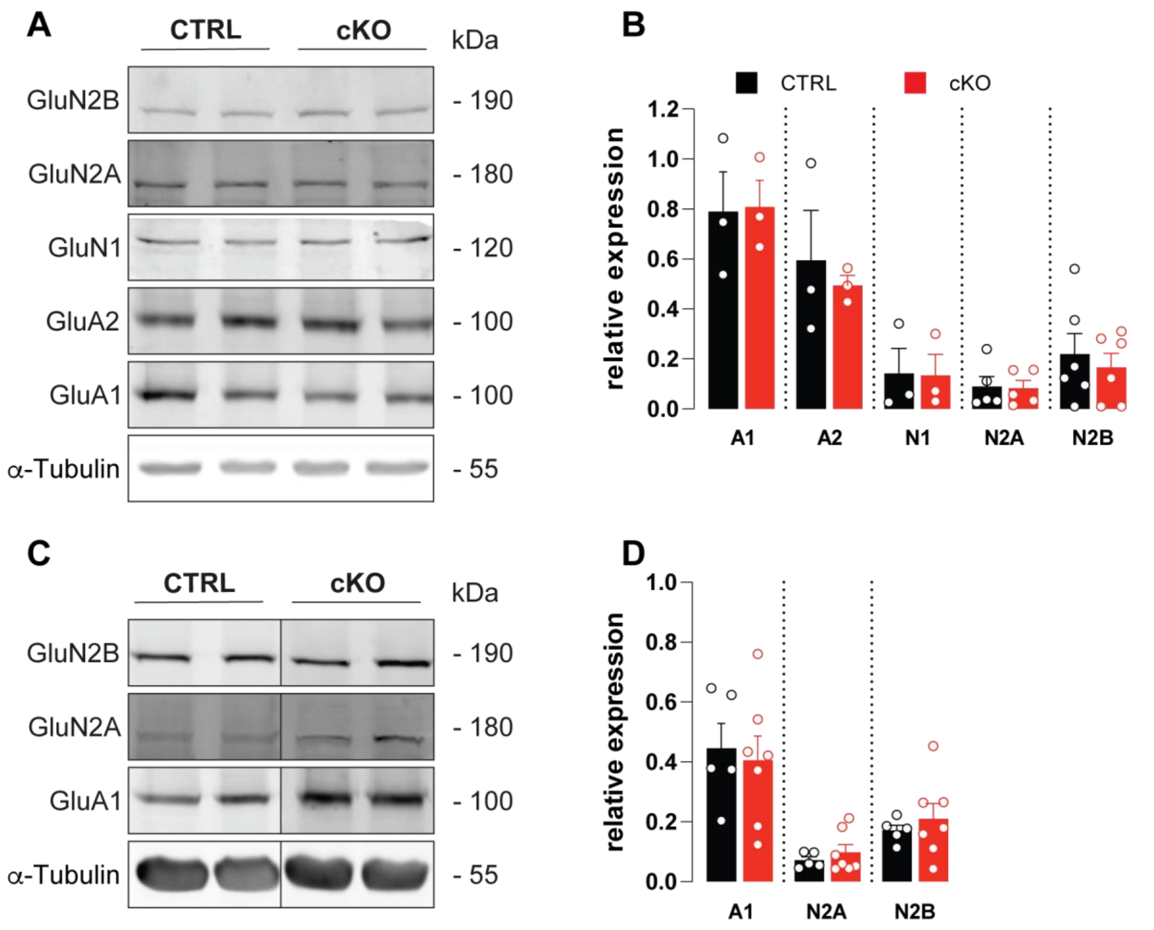

Figure S3. *AMPAR and NMDAR subunit composition is normal in cKO under basal conditions and after MWM*

(**A**) Hippocampal lysates from 8-12 weeks-old CTRL and cKO were used for immunodetection on Western blot membranes. No differences were detected in AMPAR (GluA1 N = 3 animals and GluA2 N = 3) and NMDAR (GluN1 N = 3, GluN2A N = 5, and GluN2B N = 6) subunit composition. Left: representative blots, right: densitometric quantification, normalized to loading control (α-Tubulin) on bottom.

(**B**) After completion of the MWM, hippocampal lysates from CTRL and cKO were used for immunoblotting. No difference was found in AMPAR (GluA1 N = 5-7) and NMDAR (GluN2A N = 5-7, GluN2B N = 5-7) subunit composition. Representative blots on top, densitometric quantification, normalized to loading control (α-Tubulin) on bottom.

Statistics: Unpaired Student's t test. All bar diagrams presented as means ± SEM.

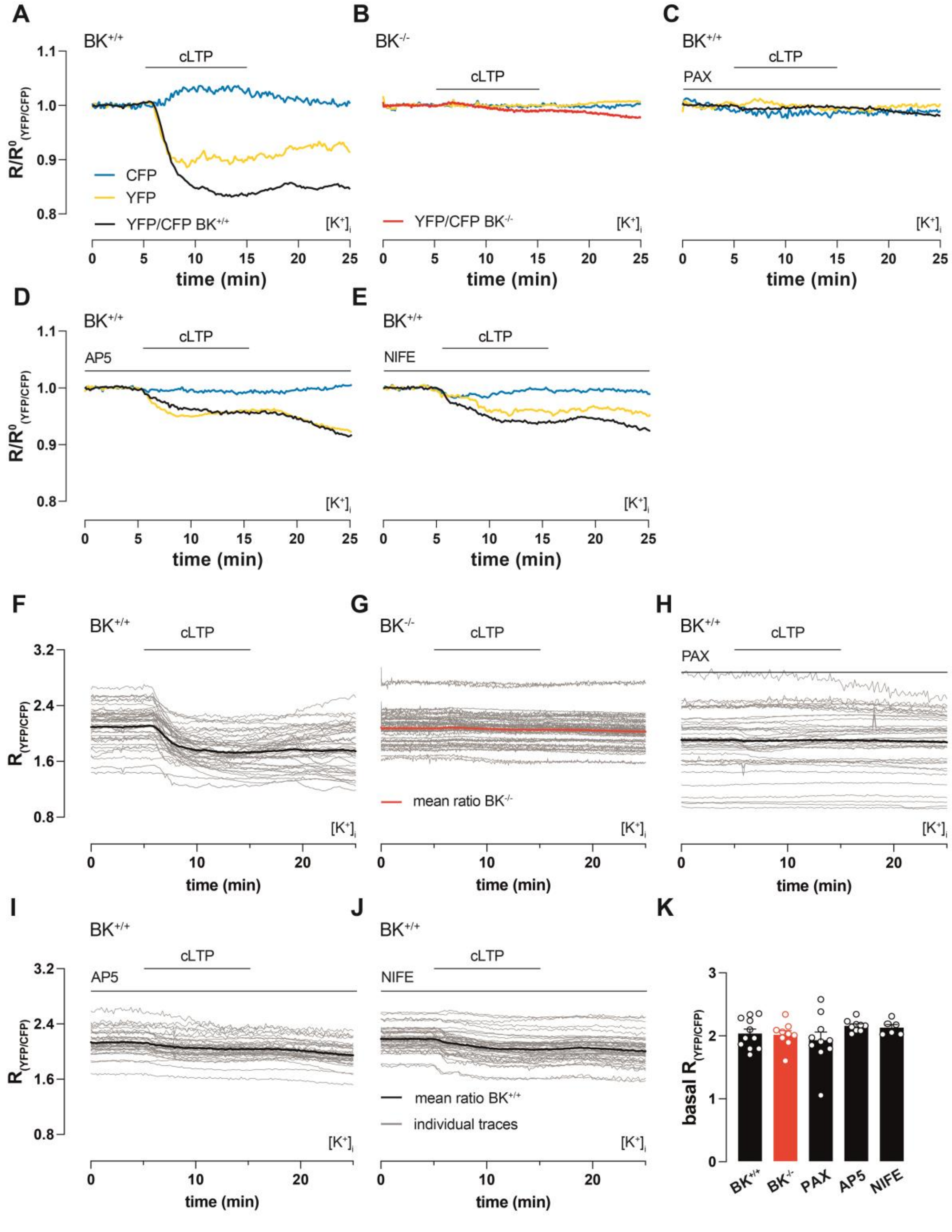

Figure S4. *Single intensities and unnormalized data from $[K^+]_i$ recordings*

(A-E) Averaged time course of ratio (YFP/CFP) are shown in black for $BK^{+/+}$ (**A, C-E**) or red for $BK^{-/-}$ **(B)**. Representative traces for single fluorophore intensities of YFP (yellow) and CFP (blue) from live K^+ imaging in Figure 4 show antiparallel progression recorded over time for (**A**) $BK^{+/+}$, (**B**) $BK^{-/-}$, (**C**) $BK^{+/+}$ with PAX, (**D**) $BK^{+/+}$ with AP5 and (**E**) $BK^{+/+}$ with NIFE.

(F-K) Data from Figure 4 C-G are shown unnormalized. 9 DIV hippocampal neuronal cultures were virally transduced at 7 DIV with the FRET-based K^+ indicator (GEPII) before cLTP induction. (**K**) Comparison of the averaged first 5 minutes of measurement from **(F)** $BK^{+/+}$ (n = 11 independent experiments from a total of n = 54 neurons obtained from N = 6 preparations), **(G)** $BK^{-/-}$ (n = 9 independent experiments from a total of n = 48 neurons obtained from N = 5 preparations) and $BK^{+/+}$ neurons inhibited by **(H)** PAX (n = 10 independent experiments from a total of n = 43 neurons obtained from N = 5 preparations), **(I)** AP5 (n = 9 independent experiments from a total of n = 37 neurons obtained from N = 5 preparations), and **(J)** NIFE (n = 6 independent experiments from a total of n = 52 neurons obtained from N = 4 preparations) did not differ, indicating comparable $[K^+]_i$ concentration. Statistics: One-way ANOVA with Tukey's multiple comparison test. All bar diagrams presented as means ± SEM.

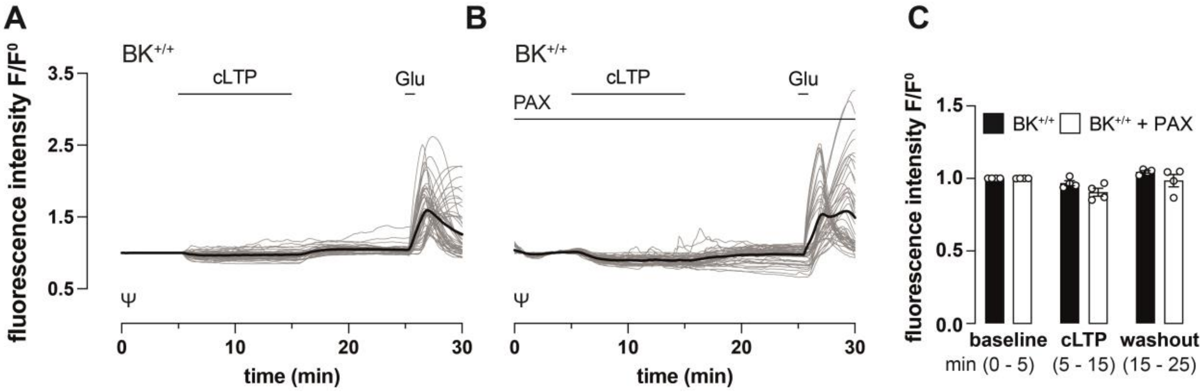

Figure S5. *Unchanged membrane potential during cLTP*

(A and B) Time course of fluorescence intensity at 493 nm in 9 DIV hippocampal neuronal cultures loaded with the potential-sensitive probe, $DiBAC_4(3)$. According to manufacturer's instruction $DiBAC_4(3)$ was present in all buffers used. Traces recorded from individual neurons from multiple experiments are plotted in grey, representative traces in black. Glutamate (20 µM) application at the end of each measurement verified cell viability and served as positive control.

(A) cLTP induction via FRP did not change membrane potential neither in $BK^{+/+}$ nor **(B)** in BK-inhibited neurons via PAX (5 µM).

(C) Comparison of normalized fluorescence intensity during baseline (first 5 min), cLTP (5 – 15 min) and washout (15 – 25 min) revealed comparable membrane potential between $BK^{+/+}$ and $BK^{+/+}$ with PAX before, during and after cLTP induction.

Statistics: Two-way ANOVA with Tukey's multiple comparison test. All bar diagrams presented as means ± SEM.

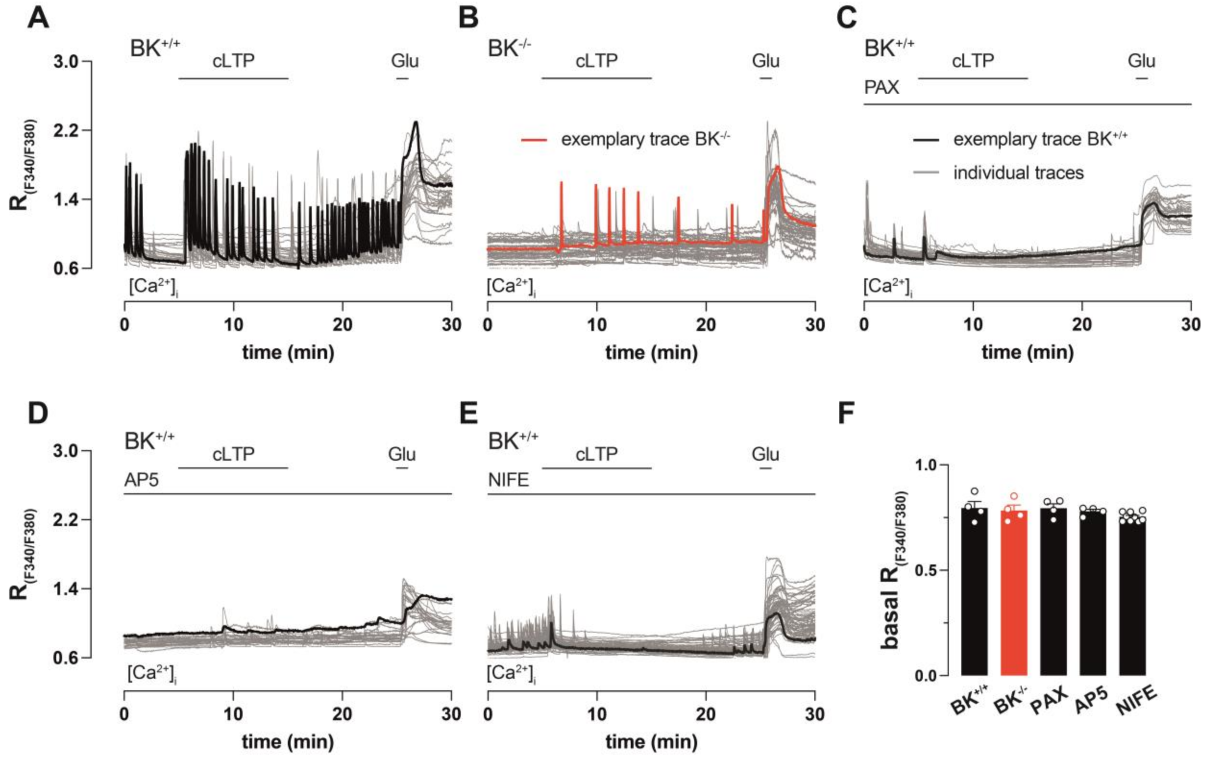

Figure S6. *Unnormalized data from [Ca^{2+}]$_i$ recordings*

(A-E) Data from Figure 5 A-E are shown unnormalized. 9 DIV hippocampal neuronal cultures loaded with the Ca^{2+}-sensitive dye Fura-2-AM. Traces from individual neurons recorded during multiple measurements are plotted in grey, representative traces in black except for **(B)** which is in red. Glutamate (20 µM) application at the end of each measurement verified cell viability and served as positive control.

(F) Comparison of the averaged first 5 minutes of measurement from **(A)** BK$^{+/+}$ (n = 4 independent experiments from a total of n = 31 neurons obtained from N = 4 preparations), **(B)** BK$^{-/-}$ (n = 4 independent experiments from a total of n = 35 neurons obtained from N = 3 preparations) and BK$^{+/+}$ neurons inhibited by **(C)** PAX (n = 4 independent experiments from a total of n = 29 neurons obtained from N = 3 preparations), **(D)** AP5 (n = 4 independent experiments from a total of n = 29 neurons obtained from N = 3 preparations), and **(E)** NIFE (n = 4 independent experiments from a total of n = 69 neurons obtained from N = 5 preparations) did not differ, indicating comparable [Ca^{2+}]$_i$ concentration between neurons.

Statistics: One-way ANOVA with Tukey's multiple comparison test. All bar diagrams presented as means ± SEM.

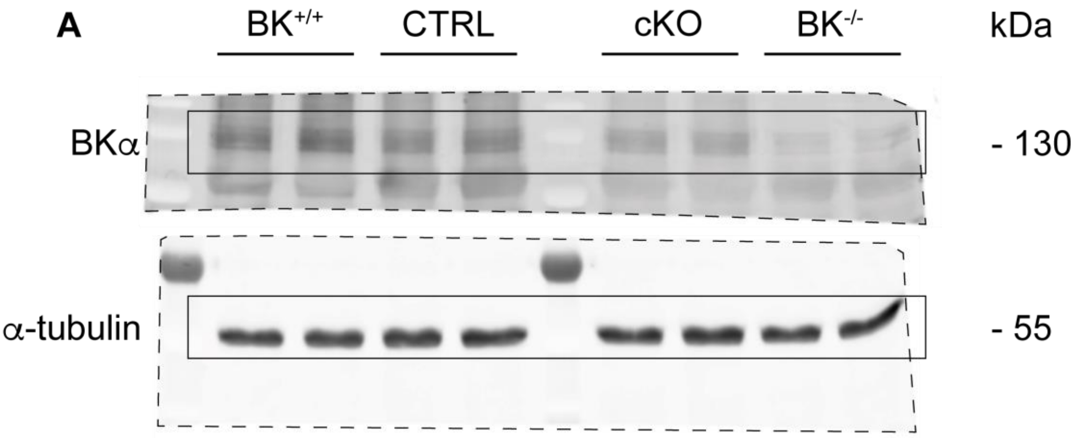

Figure S7. *Raw blot from Figure 1A*

Entire strips of Western blot membrane from Figure 1A probed with the indicated antibodies and displayed without contrast enhancement. To facilitate association to the blot shown in Figure 1, both are labeled identically.

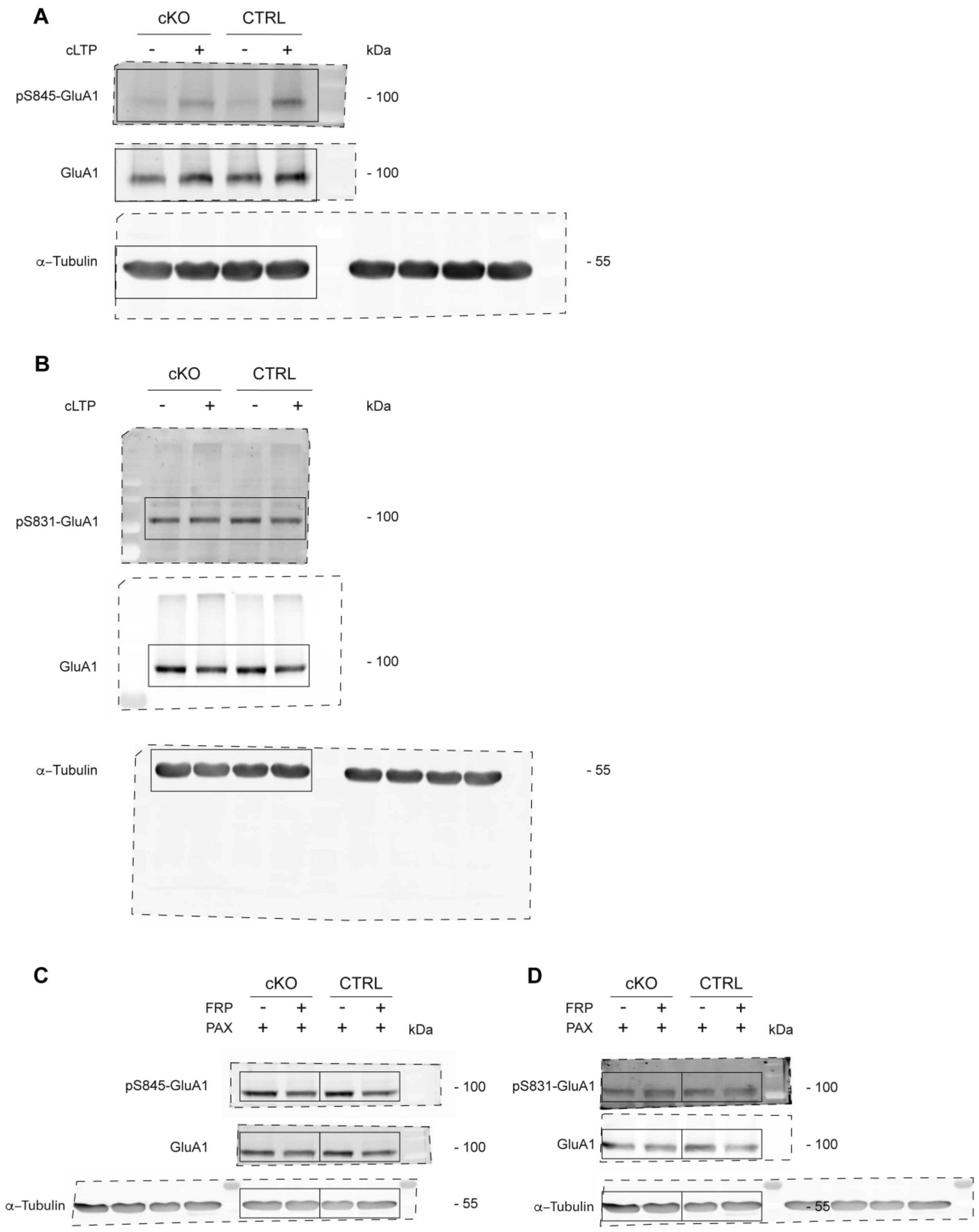

Figure S8. *Raw blots from Figure 3*

Entire strips of Western blot membranes from Figure 3 probed with the indicated antibodies and displayed without contrast enhancement. To facilitate association to the blots shown in Figure 3, panel arrangement and labelling were preserved.

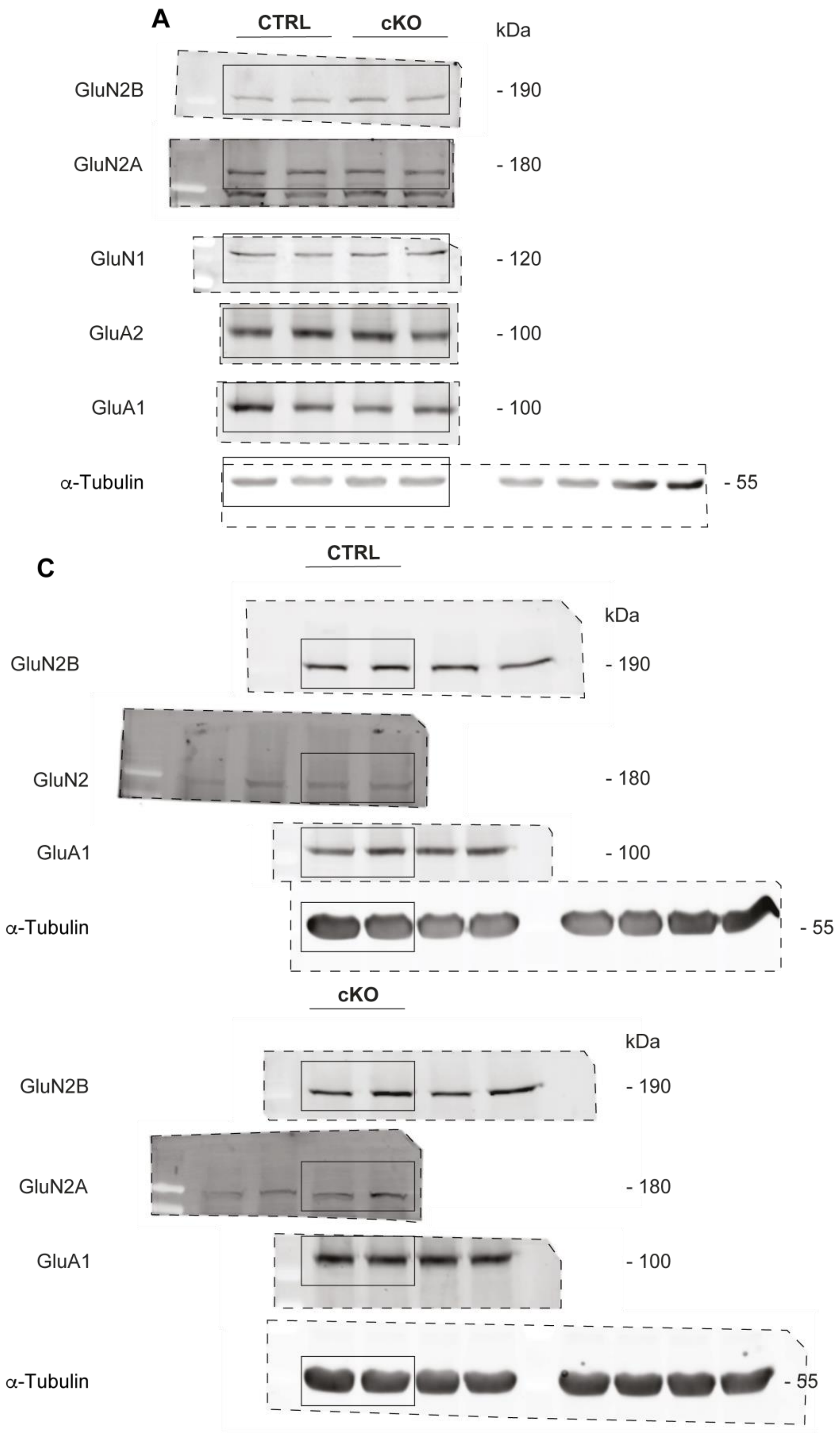

Figure S9. *Raw blots from Figure S3*

Entire strips of Western blot membranes from Figure S3 probed with the indicated antibodies and displayed without contrast enhancement. To facilitate association to the blots shown in S3, panel arrangement and labelling were preserved.
